# Tuning the Sensitivity of the *PDR5* Promoter-Based Detection of Diclofenac in Yeast Biosensors

**DOI:** 10.3390/s17071506

**Published:** 2017-06-26

**Authors:** Astrid Schuller, Gerhard Rödel, Kai Ostermann

**Affiliations:** Institute of Genetics, Technische Universität Dresden, 01062 Dresden, Germany; astrid.schuller@tu-dresden.de (A.S.); gerhard.roedel@tu-dresden.de (G.R.)

**Keywords:** diclofenac, *PDR5*, yeast, biosensor

## Abstract

The commonly used drug diclofenac is an important environmental anthropogenic pollutant. Currently, detection of diclofenac is mainly based on chemical and physical methods. Here we describe a yeast biosensor that drives the diclofenac-dependent expression of a recombinant fluorescent protein from the authentic promoter of the *PDR5* gene. This key component of the pleiotropic drug response encodes a multidrug transporter that is involved in cellular detoxification. We analyse the effects on diclofenac sensitivity of artificial *PDR5* promoter derivatives in wild-type and various yeast mutant strains. This approach enabled us to generate sensor strains with elevated drug sensitivity.

## 1. Introduction

Diclofenac monosodium (CAS 15307-79-6) is a non-steroidal anti-inflammatory drug (NSAID) that is prescribed against pain and rheumatic inflammations. Diclofenac is regarded as a priority substance in the field of European water policy [1], since it may pose a significant risk to, or via, the aquatic environment. In a long-term study of the effluents of three wastewater treatment plants (WWTP) over 10 months, diclofenac was detected with mean concentrations of 1–10 μg/L [2], whereas in a EU-wide monitoring survey of WWTP effluents a maximal concentration of 174 ng/L was detected with average values of 50 ng/L [3]. Contradictory results have been reported for the removal of diclofenac in WWTPs [4]: while in one WWTP removal rates up to 70% were found, removal was neither observed in other investigated conventional WWTPs [5] nor in a pilot sewage plant and biofilm reactors [6]. 

In the EU-wide monitoring survey cited above [3], analytics mainly encompassed chemical and physical methods as well as bioassays. For example, the yeast *Saccharomyces (S.) cerevisiae* (strain W303.1a) was used for cytotoxicity tests based on its sensitivity to oxidative stress. With cytotoxicity tests a specification of the inducing agent is not possible and bioassays capable of detecting specific substances would be desirable. As reviewed in [7], yeasts can be generated for the detection of a variety of molecules, like pharmacological compounds, odorants or intracellular metabolites. Often biosensors rely on receptor molecules, such as G-protein-coupled receptors (GPCRs), human steroid receptors or other nuclear receptors that directly, or after activation of a signal cascade, induce the transcription of a reporter construct. In the case that specific receptors for the substance to be detected are unknown, compound-responsive promoters can be used, although there is a high potential for cross-regulation. In such cases, genetic engineering approaches may lower or eliminate the undesired cross talk mediated by different transcription factors (TFs) and/or enhance the desired signal, e.g., by incorporating additional signal-specific TF binding sites.

Here we present an approach to engineer the diclofenac-responsive promoter of the *S. cerevisiae PDR5* gene that is involved in the pleiotropic drug resistance (PDR), a mechanism that enables cells to become resistant to different cytotoxic compounds. In yeast, diclofenac targets are the mitochondrial respiratory chain subunits Rip1p of complex III and Cox9p of complex IV, respectively [8]. Involved in diclofenac resistance in *S. cerevisiae* are cell wall signalling via the protein kinase C (PKC) pathway, altered zinc homeostasis and the induction of PDR [9]. Transcription of the *PDR5* gene encoding a plasma membrane ATP-binding cassette (ABC) transporter that acts as the main contributor in this PDR response is induced by diclofenac [9]. Hence the diclofenac-responsive *PDR5* promoter is an appropriate candidate for a promoter engineering approach. 

The *PDR5* promoter harbours several essential binding sites (PDREs, pleiotropic drug response elements) for Pdr1p and Pdr3p [10,11]. These transcription factors are the master regulators of the PDR and positively regulate *PDR5* expression [10,12]. Transcription of *PDR5* requires the presence of either Pdr1p or Pdr3p. When both are absent (*Δpdr1Δpdr3*), *PDR5* transcription is strongly reduced to ~2% of the WT [10]. Pdr1p and Pdr3p belong to a family of zinc cluster proteins that also includes Stb5p, Rdr1p, Yrr1p, and Pdr8p, some of which possess overlapping or identical binding sites with Pdr1p/Pdr3p [13]. Pdr1p and Pdr3p form homo- and/or heterodimers and constitute a network that cooperatively coordinates the transcriptional regulation of *PDR* genes in *S. cerevisiae* [13]. Thakur and co-workers [14] showed that Pdr1p family members can be regarded as xenobiotic receptors by directly binding structurally-diverse drugs and xenobiotics, resulting in stimulated expression of drug efflux pumps and induction of MDR (multidrug resistance, functionally analogous to the PDR). Antifungal/xenobiotic-dependent regulation of MDR additionally requires the Gal11p/MED15 subunit of the Mediator, a central coactivator that transmits regulatory signals from DNA-binding TFs to the basal transcription machinery. 

In the present work, we investigated the response of the *PDR5* wild-type (WT) promoter and of artificial promoter constructs to diclofenac. We show that deletion of the *PDR3* gene had almost no effect on diclofenac-induced *PDR5* expression, while deletion of the *PDR1* gene reduced it to almost half. Deletion of both *PDR1* and *PDR3*, however, prevented diclofenac induction of *PDR5*. Systematic deletion of PDREs in the 5′URS of *PDR5* led to the identification of a mutant version that showed a concentration-dependent response to diclofenac. It contained two PDREs, a Mot3p binding site (a TF annotated as transcriptional repressor or activator) and overlapping binding sites for additional TFs, such as Stb5p, Pdr8p and Yrr1p. Deletion of the respective TFs identified Stb5p to be important for an early response to diclofenac. Furthermore, we investigated the response of artificial reporter constructs both in WT and deletion strains of subunits of the Mediator complex. We show that increasing numbers of PDREs confer increased reporter sensitivity in the genetic background of certain deletion mutants of the Mediator subunits. 

## 2. Materials and Methods 

### 2.1. Yeast Strains 

The *S. cerevisiae* strain BY4741 (*MATa*; *his3Δ1*; *leu2Δ0*; *met15Δ0*; *ura3Δ0*) and the listed deletion strains from EUROSCARF (Supplement Table S1) were used. BY4741 is referred to as wild-type. The double deletion strain *Δpdr1Δpdr3* was generated by crossing strains *Δpdr1* (*MATa*) and *Δpdr3* (*MATα*), sporulation of selected diploid cells and subsequent tetrad analysis. Resulting strains were characterized by PCR, verifying the insertion of the kanamycin cassette into the respective gene locus. The auxotrophic markers of the generated double deletion strains were identified by growth on media with and without the respective supplements and the mating type was determined by crossing with a tester strain.

### 2.2. Growth Media and Culture Conditions

Yeast cells were cultured in YPD (2% glucose, 2% bacto-peptone, 1% yeast extract) or in synthetic defined (SD) media (2% glucose, 0.17% yeast nitrogen base without ammonium sulphate and without amino acids, 0.5% ammonium sulphate) for plasmid selection supplemented with l-histidin (60 mg/L), l-leucine (80 mg/L) and l-methionin (20 mg/L). SD basal medium was autoclaved and filter-sterilized glucose and amino acids were added before use. Diclofenac sodium salt (D6899) was obtained from Sigma-Aldrich Chemie GmbH (Steinheim, Germany). 

Stocks of diclofenac were prepared with ethanol absolute (100%) or distilled water and stored at −20 °C. Yeast cells were cultured in Erlenmeyer 50 mL flasks or 12 well plates (TPP^®^ tissue culture plates, Techno Plastic Products AG, Trasadingen, Switzerland) under shaking (180 rpm) at 30 °C.

### 2.3. Plasmid Construction and Manipulation

The multicopy yeast vector p426GPD [15] was used for the generation of all constructs. The 5′URS were cloned as a *Sac*I/*Spe*I fragment, replacing the authentic *GPD* promoter. The nucleotide sequences encoding the reporter proteins TGFP and TRFP (Evrogen Joint Stock Company, Moscow, Russia) were inserted into the *Spe*I- and *Xho*I-digested vector. Common molecular biology techniques were as described by [16]. Artificial 5′URS were synthesized by BioCat GmbH (Heidelberg, Germany). Plasmid DNA was purified with ZR Plasmid Miniprep^TM^—Classic (Zymo Research Corp., Irvine, CA, USA). The nucleotide sequence of the cloned fragments was verified by sequencing. Yeast transformation was performed using the Frozen-EZ Yeast Transformation II^TM^ (Zymo Research).

### 2.4. Response of the Yeast Cell Sensors to Diclofenac

Reporter constructs were transformed into the respective *S. cerevisiae* strain. After overnight culture, 200 μL of yeast culture (optical density (OD) at 600 nm of 0.2/0.1) containing diclofenac or solvent were added into a black 96-well plate with clear flat bottom (Brand plate pure grade^TM^ S, Ref. 781671, Brand GmbH + CO KG, Wertheim, Germany). Plates were sealed with a gas-permeable sealing membrane (“Breathe-Easy”, Diversified Biotech, Dedham, MA, USA), and cells were analysed in a FLUOstar OPTIMA (BMG Labtec, Ortenberg, Germany) under constant shaking at 30 °C for 24 h. Every hour scattered light (Nephelometric Turbidity Unit (NTU)) and relative fluorescence units (RFU) were measured with excitation filter 485 and emission filter 520 for TGFP (excitation/emission max. of 482/502 nm), and excitation filter 550 and emission filter 590 for TRFP (excitation/emission max. are 553/574 nm).

All experiments were performed as biological triplicates. Fluorescence values were normalised to a scattered light value of one (RFU/scattered light). For some experiments, normalised values of the control were subtracted from the treated ones (delta RFU/scattered light). Mean values and standard deviation (SD) were calculated using the AVERAGE and STDEV.P Excel functions. Values are shown as mean ± SD. 

### 2.5. Databases

The 5′URS sequence of *PDR5* was obtained from SGD (*Saccharomyces* Genome Database; [17]). Analysis of the 5′URS of *S. cerevisiae* was done with YEASTRACT (**Yea**st **S**earch for **T**ranscriptional **R**egulators **A**nd **C**onsensus **T**racking; [18]) and YPA (**Y**east **P**romoter **A**tlas; [19]).

## 3. Results

### 3.1. TGFP Reporter Constructs with the Native 5′URS of PDR5 Allow Detection of up to 1 μM Diclofenac

As outlined in the Introduction, transcription of the *PDR5* gene encoding the transporter Pdr5p is induced in response to diclofenac [9]. We generated reporter constructs encompassing 1000 bp of the native 5′URS of *PDR5* and TGFP, an improved variant of the green fluorescent protein, as reporter. After transformation into the WT strain BY4741, the response to different concentrations of diclofenac was evaluated (Supplement Figure S1a). Diclofenac could be detected in the concentration range of 100 to 1 μM, with indistinguishable signal intensities for 100 μM and 10 μM and high standard deviations. 

### 3.2. Diclofenac-Mediated Induction of PDR5 Depends on the Transcription Factors Pdr1p and Pdr3p, the Master Regulators of the Pleiotropic Drug Response

To design an artificial reporter construct, it was important to identify the transcription factors that are responsible for the induction. As Pdr1p and Pdr3p are described as master regulators of the PDR, we investigated the response of the reporter construct in deletion strains for these transcription factors, either as single gene deletions (*Δpdr1* and *Δpdr3*, respectively) or as a double gene deletion (*Δpdr1Δpdr3*). Upon deletion of the *PDR1* gene, the reporter construct was still inducible by diclofenac. However, the signal intensity was halved with a slight increase of the response time (Supplement Figure S1b). Deletion of *PDR3* on the other hand had no obvious influence on the signal intensities (Supplement Figure S1c), except for larger standard deviations in response to 100 μM and 10 μM diclofenac and a slightly reduced response time. Deletion of both transcription factors completely abolished induction of *PDR5* in response to diclofenac (Supplement Figure S1d). 

### 3.3. The Number of Pleiotropic Drug Response Elements (PDRE) in the WT 5′URS of the PDR5 Gene Influences the Response to Diclofenac

**Figure 1:**
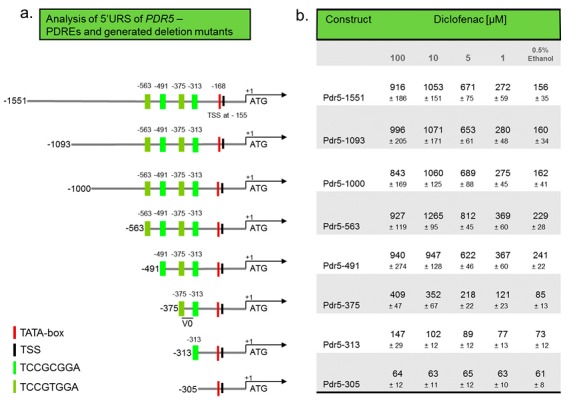
Schematic representation of a set of deletions of the 5′URS of *PDR5* (**a**) and response to different diclofenac concentrations after 24 h (**b**). Numbers on the left in (**a**) represent the number of nucleotides from the *PDR5* 5′URS.

The influence of Pdr1p and Pdr3p on the diclofenac-mediated induction of *PDR5* demonstrates the importance of the pleiotropic drug response elements (PDRE) as the binding sites of these transcription factors for the design of an artificial promoter sequence. A YEASTRACT analysis [20] of the 5′URS of *PDR5* was run to identify annotated TF binding sites, especially regarding the number and positions of PDREs. The analysis revealed 50 TFs when filtered by “*DNA binding evidence”* and “*Expression evidence”*, and four PDREs. To investigate the importance of *cis*-acting signals within the 5′URS of the *PDR5* gene for diclofenac sensitivity, we sequentially shortened the 5′URS from the 5′-end (Figure 1). 

A schematic representation of the generated constructs is shown in Figure 1a. The green bars highlight the consensus PDREs (TCCGCGGA and TCCGTGGA) and their position relative to the *PDR5* start codon. The position of the transcription start site (TSS) is taken from YPA (**Y**east **P**romoter **A**tlas) and that of the TATA-Box according to Voth et al. [21]. The longest 5′URS encompassing 1551 bp included the complete 5′ region of *PDR5* up to the flanking gene on chromosome XV, followed by deletion constructs with 1093, 1000, 563, 491, 375, 313, and 305 bp, respectively. The design of the deletion constructs from 563 onwards was related to the position of the PDREs. Starting from position 563 bp with four PDREs, each further deletion removed one additional PDRE. The shortest construct with 305 bp did not contain any PDRE. The response of the generated reporter constructs to different concentrations of diclofenac is shown in Figure 1b and Supplement Figures S2 and S3. No difference to the initially tested Pdr5-1000 construct was observed for the longer constructs 1551 and 1093. All of them allowed the detection of 5 μM and 1 μM diclofenac and showed higher induction rates with 10 μM diclofenac compared to 100 μM. Deletion construct 563, also harbouring four PDREs, yielded an even stronger signal for 10 μM diclofenac in comparison to100 μM, and higher signal intensities were noticed for 5 μM and 1 μM. Interestingly, the basal signal (without diclofenac) is also increased, similar to construct 491 with one deleted PDRE. In contrast to construct 563, the 491-reporter yielded comparable signal intensities with 100 μM and 10 μM diclofenac, but lower signals in response to 5 μM diclofenac. A further reduction of the number of PDREs in the reporter construct 375 (two PDREs) strongly reduced the signal intensities for all tested concentrations and the basal signal, not allowing the detection of 1 μM diclofenac. Downsizing the number of PDREs to one in the construct 313 further reduced the signal intensities, so that only the highest diclofenac concentration could be detected. Deletion of all PDREs in the construct 305 resulted in the absence of any diclofenac-mediated signal. In summary, the number of PDREs correlated strongly with the signal intensities obtained in response to diclofenac. However, the effects differed depending on the drug concentration and influenced the basal signal, with the highest signals in the constructs 563 and 491. 

### 3.4. The Promoter Sequence −306 to −375 Harbours Binding Sites for Mot3p, Pdr8p, Stb5p and Yrr1p

**Figure 2:**
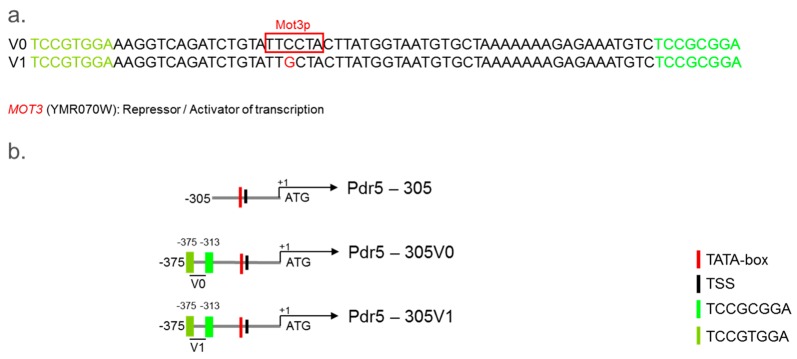
Analysis of the sequence −306 to −375 (V0) from the 5′URS of *PDR5* and of the mutated sequence (V1) and schematic presentation of generated reporter constructs. (**a**) Presentation of the sequence −306 to −375 (V0) and of the mutated sequence (V1) with a mutated Mot3p binding site; and (**b**) schematic representation of the generated deletion mutants of the 5′URS of *PDR5*, Pdr5-305 without PDREs, Pdr5-305V0 with two PDREs and a Mot3p binding site (at position −346), and Pdr5-305V1 with the mutated Mot3p binding site.

In contrast to construct 305, construct 375, with two PDREs, mediated a concentration-dependent response to diclofenac. Therefore, we analysed the DNA-sequence from position −306 to −375 in more detail with YEASTRACT (Figure 2a). 

This analysis revealed at position −346 the presence of a binding site for Mot3p, a TF annotated as transcriptional repressor or activator. Furthermore, binding sites for additional TFs (Pdr8p, Yrr1p, and Stb5p, for details see Supplement Figure S4) were identified that are either identical or overlapping with the two PDREs. All these TFs belong to the family of zinc cluster proteins and are annotated to the PDR. 

We used the DNA-sequence −306 to −375 (henceforth, named V0) for the design of an artificial diclofenac-responsive promoter. 

#### 3.4.1. Role of Mot3p for the Response of the PDR5 Promoter Sequence −306 to −375 to Diclofenac 

To investigate the role of the Mot3p binding site, we generated a variant (V1) with a mutation in the Mot3p binding site (Figure 2a and schematic presentation in Figure 2b) and analysed its effect on the diclofenac reporter constructs (Supplement Figure S5. For results for Pdr5-305 see Supplement Figure S8). The mutated Mot3p binding site led to enhanced signal intensities in response to 100 μM diclofenac in WT strains (Supplement Figure S5b), albeit associated with high standard deviations (see also Supplement Figure S6b). For 10 μM and 5 μM diclofenac only a very slight increase in the signal intensities could be detected. A comparable response was obtained in the *Δmot3* mutant (Supplement Figure S5c,d). Based on this result the V1 variant was used in further experiments to generate an artificial promoter lacking TF binding sites between the PDREs (see Section 3.6). 

#### 3.4.2. In the Absence of Stb5p the Response to Diclofenac Is Delayed 

As Stb5p, Pdr8p, and Yrr1p possess overlapping binding sites with the PDRE consensus motifs in the −306 to −375 sequence, we investigated if these TFs are involved in the response to diclofenac with the constructs Pdr5-305V0 and Pdr5-305V1 in the respective deletion strains and the WT (Supplement Figures S6a–d and S7a–f; results for Pdr5-305 see Supplement Figure S8). In the deletion mutant *Δstb5* a delayed response of both constructs to diclofenac was observed, with an even more pronounced delay for the reporter construct Pdr5-305V1. The *Δpdr8* mutant showed slightly reduced signal intensities and for the *Δyrr1* mutant, no difference to the WT was detected. Our data suggest that only Stb5p seems to be important for an early response of the *PDR5* reporter constructs. 

### 3.5. Deletion of Mediator Subunits Differentially Influence the Response Strength to Diclofenac 

**Figure 3:**
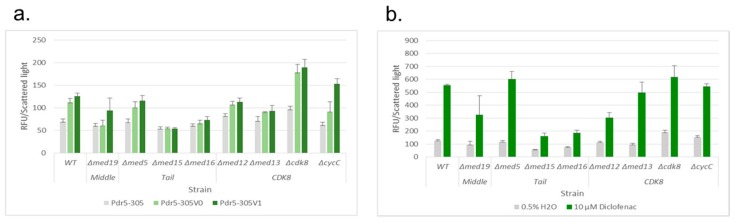
Response of Pdr5-305, Pdr5-305V0 and Pdr5-305V1 in the absence of diclofenac (**a**) and of Pdr5-305V1 in response to 10 μM diclofenac (**b**) in Wild Type (WT) cells and deletion mutants of the Mediator complex. Strains are grouped according to the corresponding Mediator module. Relative fluorescence units (RFU) divided by scattered light, Reporter: TGFP, values after 24 h.

Deletion of the Gal11p/MED15 subunit of the Mediator has been reported to prevent the xenobiotic (cycloheximide, rifampicin, ketoconazole)-induced transcription of *PDR5* [14]. We were interested whether the activation of the reporter constructs in response to diclofenac is also affected in the corresponding deletion mutant. In addition, we wanted to identify further Mediator components with critical roles in the diclofenac-induced transcription from our reporter constructs. For this approach we chose subunits of the middle, tail and CDK8 modules of the Mediator, based on the data by Lee and co-workers [22], and investigated the response of the Pdr5-305, Pdr5-305V0, and Pdr5-305V1 reporter constructs in the respective deletion mutants. We tested the response in the absence and the presence of various diclofenac concentrations and present the results with subunit assignment to the Mediator modules according to [23]. Different signal intensities were noticed for the Pdr5-305 reporter in the absence of diclofenac in the WT and the deletion mutants (Figure 3a). 

A slightly reduced response was observed in *Δmed15*, whereas the *Δcdk8* mutant exhibited a faint signal increase. The presence of two PDREs enhanced the basal response level in the WT and in nearly all deletion mutants tested, except for the *Δmed15* and the *Δmed16* mutant which showed no or a slightly increased signal, respectively. In the *Δmed19* mutant an increase of the signal was only observed for the Pdr5-305V1 reporter. The comparison of the signals obtained for Pdr5-305V0 and Pdr5-305V1 revealed an increase in the absence of the Mot3p binding site in all strains, except for *Δmed15* and *Δmed13.* In the latter mutant, no elevated signals were detected for the Pdr5-305V1 reporter. 

The response to diclofenac with the Pdr5-305V1 reporter construct is shown in Figure 3b, which was used for the design of the artificial promoters with additional PDREs. All results for Pdr5-305, Pdr5-305V0, and Pdr5-305V1 are given in the Supplement Figures S9–S11.

Strong differences were observed for the response of the reporter construct Pdr5-305V1 to diclofenac in the WT compared to the deletion mutants (Figure 3b). Signal intensities were strongly lowered in the deletion strains *Δmed15* and *Δmed16*, (subunits of the tail module), and slightly reduced in *Δmed12* (CDK8 module) and *Δmed19* (middle module). A slightly elevated signal was observed in the *Δcdk8* mutant (CDK8 module), which also showed an increased signal in the absence of diclofenac. In addition, a comparable enhancement of the signal intensity was observed in the *Δmed5* mutant (tail module) in response to 10 μM diclofenac. This effect, however, did not occur at lower diclofenac concentrations (see Supplement Figure S11). We decided to use the *Δcdk8* and *Δmed5* mutants with artificial reporter constructs in order to increase the sensitivity for diclofenac. 

### 3.6. Design of Artificial Promoter Constructs with Additional PDREs

**Figure 4:**
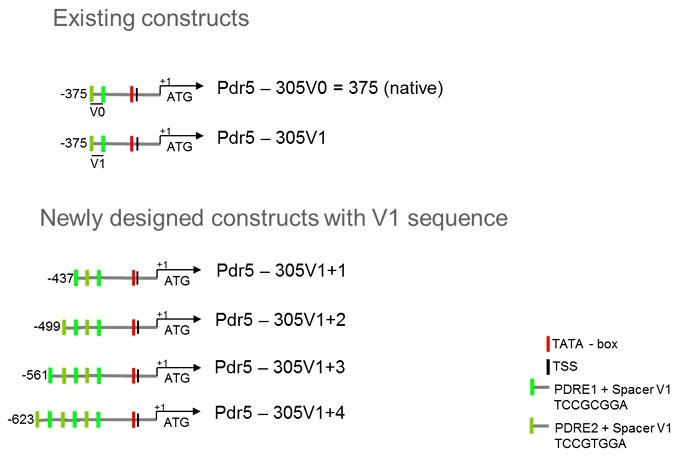
Schematic presentation of artificial promoters with spacer V1 and additional PDRE1 and PDRE2.

We designed artificial promoter constructs by alternately adding the consensus sequences PDRE1 and PDRE2 separated by the V1 spacer to the Pdr5-305V1 sequence (Figure 4).

In addition to the Pdr5-305V1 reporter with two PDREs we generated reporter constructs with three (Pdr5-305V1+1), four (Pdr5-305V1+2), five (Pdr5-305V1+3), and six PDREs (Pdr5-305V1+4) for driving the expression of TGFP or TRFP. Initial experiments with TGFP revealed increasing signal intensities with an increased number of PDREs, but indicated that the signal resolution for 0.5 μM and 0.1 μM diclofenac might be too low for revealing significant differences (see Supplement Figure S12). 

### 3.7. Response of Native and Artificial Promoter Constructs in WT, Δcdk8, and Δmed5 Strains

**Figure 5:**
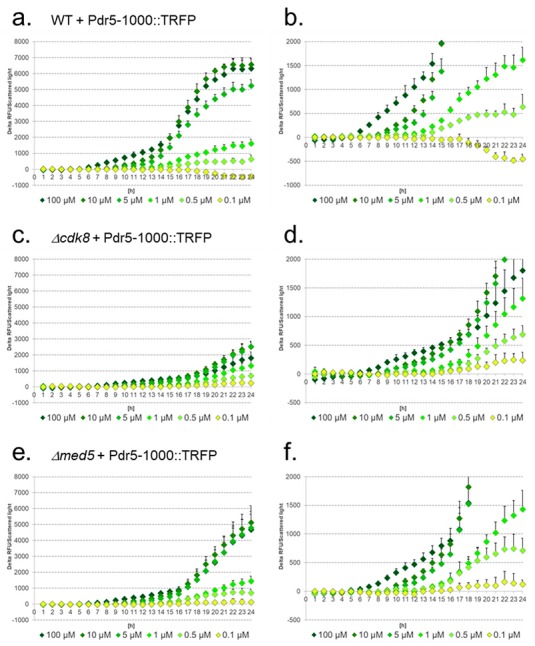
Induction of native 5′URS of *PDR5* (Pdr5-1000) in response to different diclofenac concentrations in WT (**a**,**b**), *Δcdk8* (**c**,**d**) and *Δmed5* (**e**,**f**) strains. The lower figures show enlarged sections. Delta of relative fluorescence units (RFU) divided by scattered light.

We next tested the response of the constructs controlling the expression of TRFP—which exhibits a brighter fluorescence than TGFP— in WT and *Δcdk8* and *Δmed5* mutants compared to the native 5′URS of *PDR5* (1000 bp). With the native promoter construct detection of 0.5 μM diclofenac in WT was possible (Figure 5a,b). 

While signal intensities for 100 μM and 10 μM diclofenac could not be distinguished, 5 μM, 1 μM, and 0.5 μM could be detected with decreasing signal intensities and non-overlapping standard deviations. In WT cells values (delta of the relative fluorescence units (RFU) divided by scattered light) of around 6500 for 10 μM diclofenac and 630 for 0.5 μM were reached. In the *Δcdk8* mutant even 0.1 μM diclofenac could be detected (Figure 5c,d). In contrast, 100 μM, 10 μM, 5 μM, and 1 μM could not be distinguished, and overlapping standard deviations with the signals for 1 μM diclofenac prevented the significance of the detection of 0.5 μM. Signal intensities for 100 μM were lower compared to 10 μM and 5 μM and indistinguishable from 1 μM. The highest delta RFU/scattered light values reached around 2500. For 0.5 μM diclofenac, values of approx. 680 and for 0.1 μM of ca. 230 were obtained. In the *Δmed5* strain (Figure 5e,f), again 100 μM, 10 μM and 5 μM could not be distinguished, and in addition also the standard deviations for delta RFU/scattered light values obtained for 1 μM and 0.5 μM were overlapping at nearly all time-points investigated. A faintly increased signal with high standard deviation was detected for 0.1 μM diclofenac. The highest obtained Delta RFU/Scattered light values reached around 5000. For 0.5 μM and 0.1 μM diclofenac, values of ca. 700 and ca. 120, respectively, were obtained.

**Figure 6:**
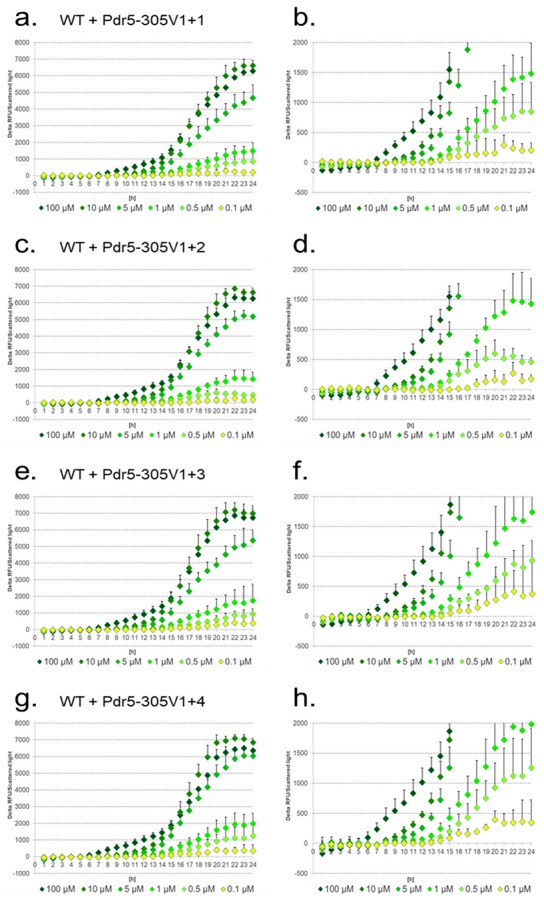
Induction of the artificial reporter constructs with additional PDREs in response to different diclofenac concentrations in the WT. The lower figures show enlarged sections. Pdr5-305V1+1 (**a**,**b**); Pdr5-305V1+2 (**c**,**d**); Pdr5-305V1+3 (**e**,**f**); Pdr5-305V1+4 (**g**,**h**). Delta of relative fluorescence units (RFU) divided by scattered light.

We next tested the artificial promoter constructs in the WT (Figure 6). 

The signal intensities of the reporter constructs slightly increased from 0.1 μM up to 5 μM diclofenac by the stepwise addition of PDREs. The slightest change was observed between the reporter constructs Pdr5-305V1+1 and Pdr5-305V1+2 (Figure 6a–d), although one additional PDRE (Pdr5-305V1+2) slightly enhanced the response to 5 μM diclofenac, while reducing the signals for 0.5 μM. It is important to note, that Pdr5-305V1+2 possesses the same number of PDREs as the native 5′URS of *PDR5* (Pdr5-1000). In contrast to the authentic 5′URS (see Figure 5a,b), 0.1 μM diclofenac is detectable with the artificial construct, although with high standard deviations. Adding additional PDREs increased the signal intensities in response to low concentrations but with very high, overlapping standard deviations (Figure 6e–h). 

**Figure 7:**
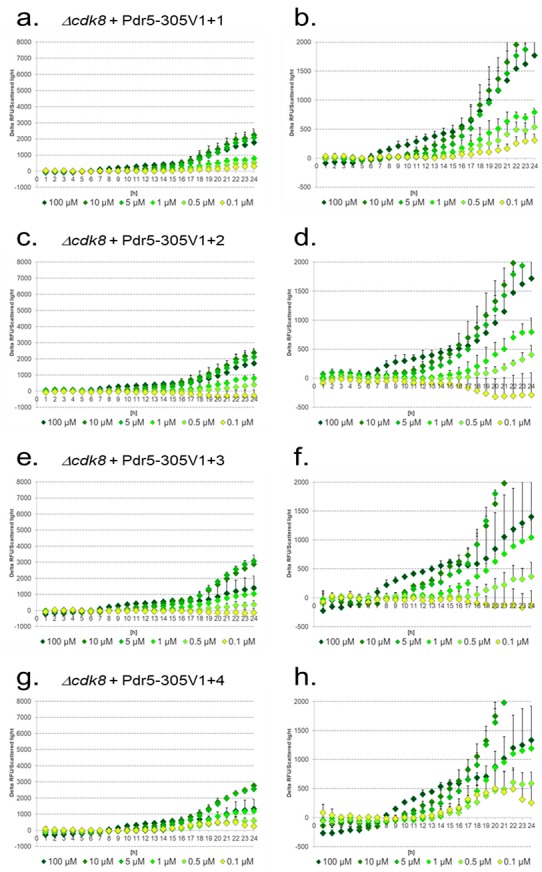
Induction of the artificial reporter constructs with additional PDREs in response to different diclofenac concentrations in the *Δcdk8* mutant. The lower figures show enlarged sections. Pdr5-305V1+1 (**a**,**b**); Pdr5-305V1+2 (**c**,**d**); Pdr5-305V1+3 (**e**,**f**); Pdr5-305V1+4 (**g**,**h**). Delta of relative fluorescence units (RFU) divided by scattered light.

In the *Δcdk8* mutant a complete different response (Figure 7) was observed. 

Overall signal intensities were low and additional PDREs resulted in a strong imbalance in the response to different diclofenac concentrations. One-hundred micromoles of diclofenac was detected with lower signal intensities as 10 μM and 5 μM, and additional PDREs resulted in a comparable signal strength for 1 μM and 100 μM diclofenac (Figure 7e–h). With one additional PDRE (Figure 7a,b), 0.1 μM diclofenac could be detected with overlapping standard deviations to 0.5 μM. Interestingly, with one or two more PDREs, 0.1 μM could not be detected and negative values were obtained (Figure 7c–f). Addition of four PDREs again allowed positive signals for 0.1 μM diclofenac but the mean values were nearly indistinguishable from those for 0.5 μM diclofenac (Figure 7g,h).

**Figure 8:**
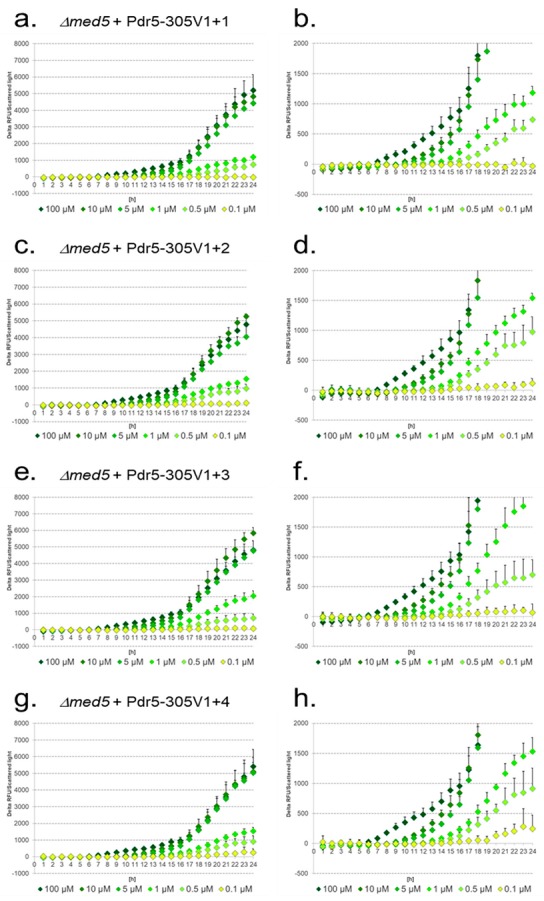
Induction of the artificial reporter constructs with additional PDREs in response to different diclofenac concentrations in the *Δmed5* mutant. The lower figures show enlarged sections. Pdr5-305V1+1 (**a**,**b**); Pdr5-305V1+2 (**c**,**d**); Pdr5-305V1+3 (**e**,**f**); Pdr5-305V1+4 (**g**,**h**). Delta of relative fluorescence units (RFU) divided by scattered light.

In the *Δmed5* mutant, overall signal intensities were slightly lower compared to WT, with lower standard deviations for the values in response to lower diclofenac concentrations (Figure 8). 

Signals for 5 μM could not be distinguished from those for 100 μM and 10 μM diclofenac. Additional PDREs enhanced the signal intensities in response to lower diclofenac concentrations. For 1 μM diclofenac a strongly enhanced signal is obtained up to the addition of three more PDREs (Pdr5-305V1+3) (Figure 8a–f). Addition of one more PDRE resulted in a decreased signal for 1 μM diclofenac as observed with the reporter Pdr5-305V1+4 (Figure 8g,h). For 0.5 μM diclofenac, an increased signal is observed up to the addition of two PDREs (Pdr5-305V1+2) (Figure 8a–d). The addition of one more PDRE (Pdr5-305V1+3) (Figure 8e–f) reduced this signal and addition of two more PDREs (Pdr5-305V1+4) (Figure 8g–h) enhanced it again to the values obtained with Pdr5-305V1+2 (Figure 8c,d). In contrast to these results, the signal intensities for 0.1 μM showed a constant increase with additional PDREs (Figure 8a–h). Exploiting the Pdr5-305V1+4 reporter and the *Δmed5* mutant, even 0.1 μM diclofenac could readily be detected, and 0.5 μM could be distinguished from 1 μM diclofenac. 

## 4. Discussion

As outlined in the Introduction, the *PDR5* promoter responds to the presence of diclofenac. In order to reveal the *cis*-acting elements and *trans*-acting factors that mediate this response, a reporter construct consisting of 1000 bp of the native 5′URS of *PDR5* fused to TGFP was generated. The role of the Zn(II)_2_Cys_6_ zinc finger regulators Pdr1p and Pdr3p, which are known to be essential regulators of the pleiotropic drug response [24], was analysed by introducing the reporter construct into yeast mutant strains lacking either *PDR3* or *PDR1*, or both *PDR3* and *PDR1*. While the deletion of *PDR3* had almost no effect and deletion of *PDR1* diminished the signal by 50%, the absence of both genes completely abolished the response to diclofenac, in line with previous data [10]. The observation that Pdr3p is apparently not involved in the diclofenac-induced transcription under our experimental settings underlines the notion that the specific functions of Pdr1p and Pdr3p are not entirely redundant [25,26]. It has been shown that mitochondria-dependent PDR effects require an increased expression of *PDR3* and are independent of *PDR1* [27]. The induced *PDR5* transcription in response to loss of mitochondrial DNA is strictly dependent on Pdr3p [28]. As Pdr1p is the only zinc cluster protein in a network of TFs, including Pdr3p, Stb5p, Yrr1p, Yrm1p (paralog of Yrr1p) and Rdr1p, that is able to form heterodimers with more than one partner [13], it was proposed that it may act in fine tuning the regulation of multidrug resistance genes [29] by recruiting other zinc cluster proteins. Interestingly, our data obtained with a *STB5* deletion strain indicate that Stb5p may play a role in the early response to diclofenac. 

Furthermore, we characterized the role of the zinc-finger protein Mot3p on the diclofenac-mediated response of the reporter construct. Mot3p is involved either as an activator or a repressor in diverse pathways, e.g., in mating pheromone signalling [30] or osmostress response [31]. Only recently it was shown that Mot3p participates in the regulation of *RTA1*, a gene involved in PDR [32]. We show that both, a mutated Mot3p binding site in the *PDR5* promoter and deletion of the *MOT3* gene, result in increased signals, indicating that Mot3p apparently represses our reporter constructs.

Our results on deletion mutants of the 5′-flanking region of *PDR5* (except for the deletion mutants −563 and −491) are in line with the data of Katzmann and co-worker [11], according to which Pdr1p/Pdr3p are the key activating factors of *PDR5* transcription. Each of the PDREs seems to contribute equally to the *PDR5* expression level. This is also in agreement with our results for the Pdr5-305, Pdr5-305V0, and Pdr5-305V1 reporter constructs in WT and in deletion strains of Mediator subunits, which showed enhanced signals by introducing additional PDREs.

Promoter engineering by introducing additional TF binding sites to modulate the reporter output is a widely-used approach (for a recent review see [33]). For example, the addition of multiple copies of UAS (upstream activating sequences) to core promoters enhanced the transcriptional output in *Yarrowia lipolytica* [34,35].

Our results underline the importance of the Mediator which, in *S. cerevisiaei*, comprises 25 subunits that are arranged in the head, middle, tail, and kinase module [23]. As a central coactivator, Mediator is linking activators with the basal transcription machinery and is required for regulated transcription by RNA polymerase (Pol) II in all eukaryotes. Pdr1p family members in *S. cerevisiae* directly bind structurally-diverse drugs and xenobiotics by a discrete ligand binding domain, resulting in the stimulated expression of drug efflux pumps [14]. It was shown by Thakur and co-workers [14] that Pdr1p physically and functionally interacts with the Gal11p/MED15 subunit of the Mediator and that deletion of this Mediator subunit prevent the xenobiotic (cycloheximide, rifampicin, ketoconazole)-induced transcription of *PDR5*. We obtained comparable results for the induction by diclofenac of PDR5-305V0 and PDR5-305V1 reporter constructs (harbouring two PDREs) in the *Δmed15* mutant strain compared with the WT. In addition, we obtained strongly-reduced induction rates with these reporter constructs in the *Δmed16* mutant strain.

Our data document that engineering of the *PDR5* promoter is a powerful tool to generate reporter constructs that mediate varying sensitivity to diclofenac. Specifically, the construct PDR5-305V1+4, exhibiting six PDRE sites, can be a promising candidate on the way towards a yeast-based biosensor that allows the detection of diclofenac in wastewater. In this context it may be worth testing whether the construct exhibits a higher sensitivity in the background of mutant yeast strains affected in the efflux or uptake of diclofenac. Fine-tuning can be achieved by deletions of specific Mediator subunits in the reporter strains. Mutagenesis of involved TFs and their binding sites may also be a strategy to generate the next generation of diclofenac biosensors exhibiting a higher sensitivity and specificity, as recently shown for the generation of a bisphenol A-specific yeast bioreporter [36]. 

## Supplementary Materials

The following are available online at http://www.mdpi.com/1424-8220/17/7/1506/s1, Table S1: *S. cerevisiae* strains used in this study. Figure S1: Induction of native *PDR5* 5′URS (1000 bp) in response to different diclofenac concentrations. Figure S2: Response of the generated deletion mutants (1551–563) to different diclofenac concentrations in BY4741 (WT). Figure S3: Response of the generated deletion mutants (491–305) to different diclofenac concentrations in BY4741 (WT). Figure S4: Result of YEASTRACT analysis of the sequence −306 to −375 (V0) of the native 5′URS of *PDR5* and of the generated sequence (V1) without a Mot3p binding site. Figure S5: Induction of Pdr5-305V0 and Pdr5-305V1 in response to different diclofenac concentrations in BY4741 (WT) and *Δmot3*. Figure S6: Induction of Pdr5-305V0 and Pdr5-305V1 in response to different diclofenac concentrations in WT and *Δstbt5* strains. Figure S7: Induction of Pdr5-305V0 and Pdr5-305V1 in response to different diclofenac concentrations in WT, *Δpdr8*, and *Δyrr1*. Figure S8: Response of Pdr5-305 to different diclofenac concentrations in BY4741 (WT) and in the deletion strains *Δmot3*, *Δstb5*, *Δpdr8*, and *Δyrr1*. Figure S9: Induction of Pdr5-305 in response to different concentrations of diclofenac in WT cells and deletion mutants of the Mediator complex. Figure S10: Induction of Pdr5-305V0 in response to different concentrations of diclofenac in WT cells and deletion mutants of the Mediator complex. Figure S11: Induction of Pdr5-305V1 in response to different concentrations of diclofenac in WT cells and deletion mutants of the Mediator complex. Figure S12: Induction of Pdr5-305, Pdr5-305V1, Pdr5-305V1+1, and Pdr5-305V1+3 in response to different diclofenac concentrations in WT cells. Reporter: TGFP.

## Abbreviations

PDR	Pleiotropic Drug Response
PDRE	Pleiotropic Drug Response Element
TF	Transcription Factor
URS	Upstream Regulatory Sequence

## References

[1] European Parliament, Council of the European Union (2013). Directive 2013/39/EU of the European Parliament and of the Council of 12 August 2013 amending Directives 2000/60/EC and 2008/105/EC as regards priority substances in the field of water policy. Off. J. Eur. Union.

[2] Reemtsma T., Weis S., Mueller J., Petrovic M., Gonzáles S., Barcelo D., Ventura F., Knepper T.P. (2006). Polar pollutants entry into the water cycle by municipal wastewater: A european perspective. Environ. Sci. Technol..

[3] Loos R., Carvalho R., António D.C., Comero S., Locoro G., Tavazzi S., Paracchini B., Ghiani M., Lettier T., Blaha L. (2013). EU-wide monitoring survey on emerging polar organic contaminants in wastewater treatment plant effluents. Water Res..

[4] Heberer H. (2002). Occurrence, fate, and removal of pharmaceutical residues in the aquatic environment: A review of recent research data. Toxicol. Lett..

[5] Clara M., Strenn B., Gans O., Martinez E., Kreuzinger N., Kroiss H. (2005). Removal of selected pharmaceuticals, fragrances and endocrine disrupting compounds in a membrane bioreactor and conventional wastewater treatment plants. Water Res..

[6] Zwiener C., Frimmel F.H. (2003). Short-term tests with a pilot sewage plant and biofilm reactors for the biological degradation of the pharmaceutical compounds clofibric acid, ibuprofen, and diclofenac. Sci. Total Environ..

[7] Adeniran A., Sherer M., Tyo K.E.J. (2015). Yeast-based biosensors: Design and applications. FEMS Yeast Res..

[8] Van Leeuwen J.S., Orij R., Luttik M.A.H., Smits G.J., Vermeulen N.P.E., Vos J.C. (2011). Subunits Rip1p and Cox9p of the respiratory chain contribute to diclofenac-induced mitochondrial Dysfunction. Microbiology.

[9] Van Leeuwen J.S., Vermeulen N.P.E., Vos J.C. (2011). Involvement of the Pleiotropic Drug Resistance Response, Protein Kinase C Signaling, and Altered Zinc Homeostasis in Resistance of *Saccharomyces cerevisiae* to diclofenac. Appl. Environ. Microbiol..

[10] Katzmann D.J., Burnett P.B., Golin J., Mahe Y., Moye-Rowley W.S. (1994). Transcriptional Control of the Yeast *PDR5* Gene by the *PDR3* Gene Product. Mol. Cell. Biol..

[11] Katzmann D.J., Hallstrom T.C., Mahe Y., Moye-Rowley W.S. (1996). Multiple Pdr1p/Pdr3p binding sites are essential for normal expression of the ATP binding cassette transporter protein-encoding Gene *PDR5*. J. Biol. Chem..

[12] Balzi E., Wang M., Leterme S., Van Dyck L., Goffe A. (1994). *PDR5*, a Novel Yeast multidrug resistance conferring transporter controlled by the transcription regulator *PDR1*. J. Biol. Chem..

[13] MacPherson S., Larochelle M., Turcotte B. (2006). A Fungal Family of Transcriptional Regulators: The Zinc Cluster Proteins. Microbiol. Mol. Biol. Rev.

[14] Thakur J.K., Arhanari H., Yang F., Pan S.J., Fan X., Breger J., Frueh D., Gulshan K., Li D.K., Mylonakis E. (2008). A nuclear receptor-like pathway regulating multidrug resistance in fungi. Nature.

[15] Mumberg D., Muller R., Funk M. (1995). Yeast vectors for the controlled expression of heterologous proteins in different genetic backgrounds. Gene.

[16] Ausubel F.M., Brent R., Kingston R.E., Moore D.D., Seidman J.G., Smith J.A., Struhl K. (1987). Current Protocols in Molecular Biology.

[17] Saccharomyces Genome Database. http://www.yeastgenome.org/.

[18] YEASTRACT. http://www.yeastract.com/.

[19] YPA (Yeast Promoter Atlas). http://ypa.csbb.ntu.edu.tw/.

[20] Teixeira M.C., Monteiro P.T., Guerreiro J.F., Gonc J.P., Mira N.P., Costa dos Santos S., Cabrito T.R., Palma M., Costa C., Francisco A.P. (2014). The YEASTRACT database: An upgraded information system for the analysis of gene and genomic transcription regulation in *Saccharomyces cerevisiae*. Nucleic Acids Res..

[21] Voth W.P., Takahata S., Nishikawa J.L., Metcalfe B.M., Näär A.M., Stillman D.J. (2014). A Role for FACT in Repopulation of Nucleosomes at Inducible Genes. PLoS ONE.

[22] Lee S.K., Fletcher A.G.L., Zhang L., Chen X., Fischbeck J.A., Stargell L.A. (2010). Activation of a Poised RNAPII-Dependent Promoter Requires Both SAGA and Mediator. Genetics.

[23] Plaschka C., Nozawa K., Cramer P. (2016). Mediator architecture and RNA polymerase II interaction. J. Mol. Biol..

[24] Paul S., Moye-Rowley W.S. (2014). Multidrug resistance in fungi: Regulation of transporter-encoding gene expression. Front. Physiol..

[25] Kolaczkowska A., Goffeau A. (1999). Regulation of pleiotropic drug resistance in yeast. Drug Resist. Updates.

[26] DeRisi J., van den Hazel B., Marc P., Balzi E., Brown P., Jacq C., Goffeau A. (2000). Genome microarray analysis of transcriptional activation in multidrug resistance yeast mutants. FEBS Lett..

[27] Devaux F., Carvajal E., Moye-Rowley S., Jacq C. (2001). Genome-wide studies on the nuclear *PDR3*-controlled response to mitochondrial dysfunction in yeast. FEBS Lett..

[28] Hallstrom T.C., Moye-Rowley W.S. (2000). Multiple Signals from Dysfunctional Mitochondria Activate the Pleiotropic Drug Resistance Pathway in *Saccharomyces cerevisiae*. J. Biol. Chem..

[29] Akache B., MacPherson S., Sylvain M.A., Turcotte B. (2004). Complex Interplay among Regulators of Drug Resistance Genes in *Saccharomyces cerevisiae*. J. Biol. Chem..

[30] Grishin A.V., Rothenberg M., Downs M.A., Blumer K.J. (1998). Mot3, a Zn Finger Transcription Factor That Modulates Gene Expression and Attenuates Mating Pheromone Signaling in *Saccharomyces cerevisiae*. Genetics.

[31] Martínez-Montañés F., Rienzo A., Poveda-Huertes D., Pascual-Ahuir A., Proft M. (2013). Activator and Repressor Functions of the Mot3 Transcription Factor in the Osmostress Response of *Saccharomyces cerevisiae*. Eukaryot. Cell.

[32] Kołaczkowska A., Manente M., Kołaczkowski M., Laba J., Ghislain M., Wawrzycka D. (2012). The regulatory inputs controlling pleiotropic drug resistance and hypoxic response in yeast converge at the promoter of the aminocholesterol resistance gene *RTA1*. FEMS Yeast Res..

[33] Deaner M., Alper H.S. (2016). Promoter and Terminator Discovery and Engineering. Adv. Biochem. Eng. Biotechnol..

[34] Blazeck J., Liu L., Redden H., Alper H. (2011). Tuning Gene Expression in *Yarrowia lipolytica* by a Hybrid Promoter Approach. Appl. Environ. Microbiol..

[35] Blazeck J., Reed B., Garg R., Gerstner R., Pan A., Agarwal V., Alper H.S. (2012). Generalizing a hybrid synthetic promoter approach in *Yarrowia lipolytica*. Appl. Microbiol. Biotechnol..

[36] Rajasärkkä J., Virta M. (2013). Characterization of a Bisphenol A Specific Yeast Bioreporter Utilizing the Bisphenol A-Targeted Receptor. Anal. Chem..

