# Supplementary material for: Tuning the Sensitivity of the PDR5 Promoter-Based Detection of Diclofenac in Yeast Biosensors
Source: Sensors (Basel). 2017 Jun 26;17(7):1506. doi: 10.3390/s17071506 (PMC5539612; doi:10.3390/s17071506)
Supplement: Supplementary file 1 [file sensors-17-01506-s001.zip › Supplement_Resubmission 200617.pptx]

## Slide 1
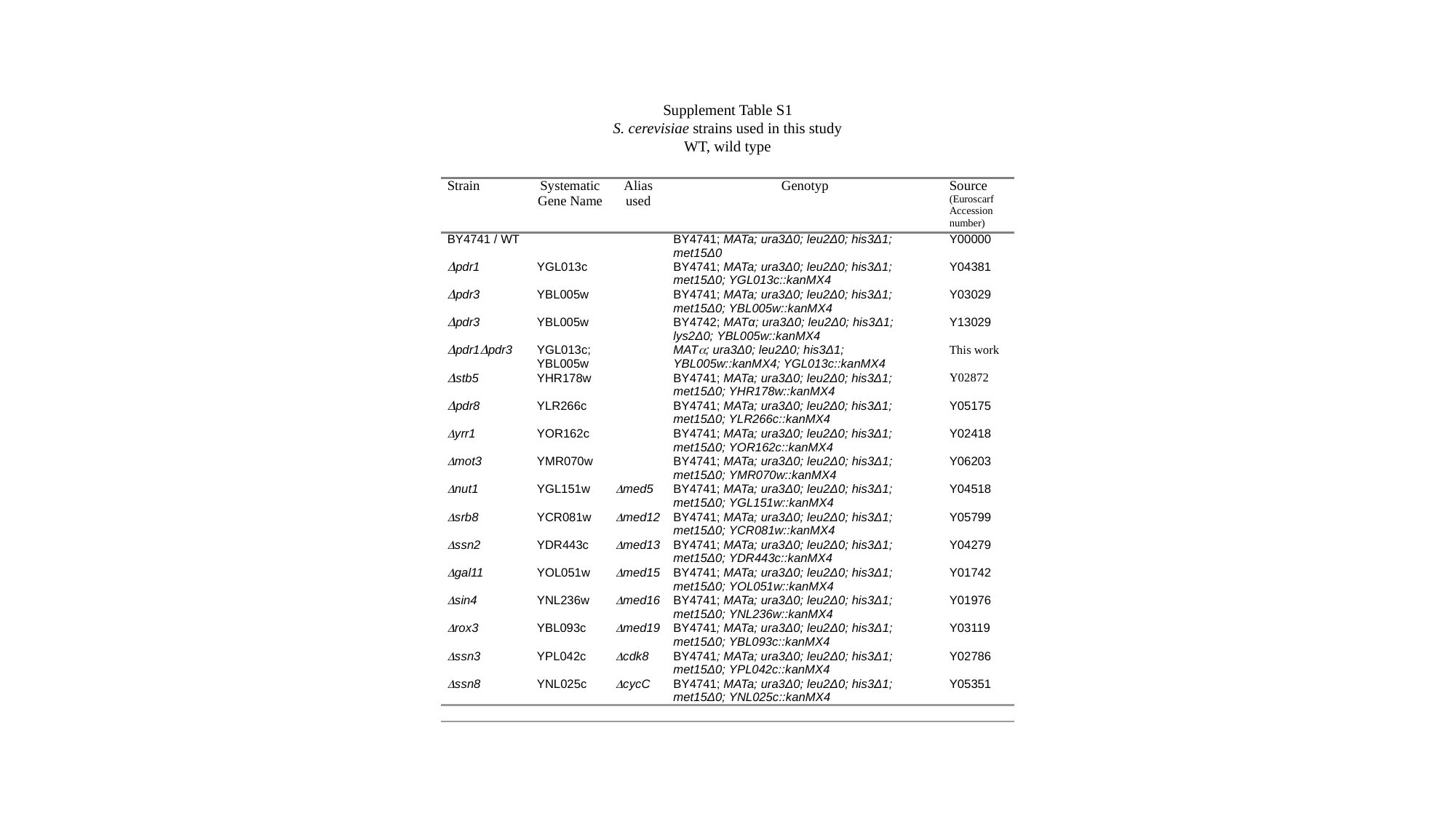

Supplement Table S1
S. cerevisiae strains used in this study
WT, wild type
| Strain | Systematic Gene Name | Alias used | Genotyp | Source (Euroscarf Accession number) |
| --- | --- | --- | --- | --- |
| BY4741 / WT | | | BY4741; MATa; ura3Δ0; leu2Δ0; his3Δ1; met15Δ0 | Y00000 |
| Dpdr1 | YGL013c | | BY4741; MATa; ura3Δ0; leu2Δ0; his3Δ1; met15Δ0; YGL013c::kanMX4 | Y04381 |
| Dpdr3 | YBL005w | | BY4741; MATa; ura3Δ0; leu2Δ0; his3Δ1; met15Δ0; YBL005w::kanMX4 | Y03029 |
| Dpdr3 | YBL005w | | BY4742; MATα; ura3Δ0; leu2Δ0; his3Δ1; lys2Δ0; YBL005w::kanMX4 | Y13029 |
| Dpdr1Dpdr3 | YGL013c; YBL005w | | MATa; ura3Δ0; leu2Δ0; his3Δ1; YBL005w::kanMX4; YGL013c::kanMX4 | This work |
| Dstb5 | YHR178w | | BY4741; MATa; ura3Δ0; leu2Δ0; his3Δ1; met15Δ0; YHR178w::kanMX4 | Y02872 |
| Dpdr8 | YLR266c | | BY4741; MATa; ura3Δ0; leu2Δ0; his3Δ1; met15Δ0; YLR266c::kanMX4 | Y05175 |
| Dyrr1 | YOR162c | | BY4741; MATa; ura3Δ0; leu2Δ0; his3Δ1; met15Δ0; YOR162c::kanMX4 | Y02418 |
| Dmot3 | YMR070w | | BY4741; MATa; ura3Δ0; leu2Δ0; his3Δ1; met15Δ0; YMR070w::kanMX4 | Y06203 |
| Dnut1 | YGL151w | Dmed5 | BY4741; MATa; ura3Δ0; leu2Δ0; his3Δ1; met15Δ0; YGL151w::kanMX4 | Y04518 |
| Dsrb8 | YCR081w | Dmed12 | BY4741; MATa; ura3Δ0; leu2Δ0; his3Δ1; met15Δ0; YCR081w::kanMX4 | Y05799 |
| Dssn2 | YDR443c | Dmed13 | BY4741; MATa; ura3Δ0; leu2Δ0; his3Δ1; met15Δ0; YDR443c::kanMX4 | Y04279 |
| Dgal11 | YOL051w | Dmed15 | BY4741; MATa; ura3Δ0; leu2Δ0; his3Δ1; met15Δ0; YOL051w::kanMX4 | Y01742 |
| Dsin4 | YNL236w | Dmed16 | BY4741; MATa; ura3Δ0; leu2Δ0; his3Δ1; met15Δ0; YNL236w::kanMX4 | Y01976 |
| Drox3 | YBL093c | Dmed19 | BY4741; MATa; ura3Δ0; leu2Δ0; his3Δ1; met15Δ0; YBL093c::kanMX4 | Y03119 |
| Dssn3 | YPL042c | Dcdk8 | BY4741; MATa; ura3Δ0; leu2Δ0; his3Δ1; met15Δ0; YPL042c::kanMX4 | Y02786 |
| Dssn8 | YNL025c | DcycC | BY4741; MATa; ura3Δ0; leu2Δ0; his3Δ1; met15Δ0; YNL025c::kanMX4 | Y05351 |
| | | | | |

## Slide 2
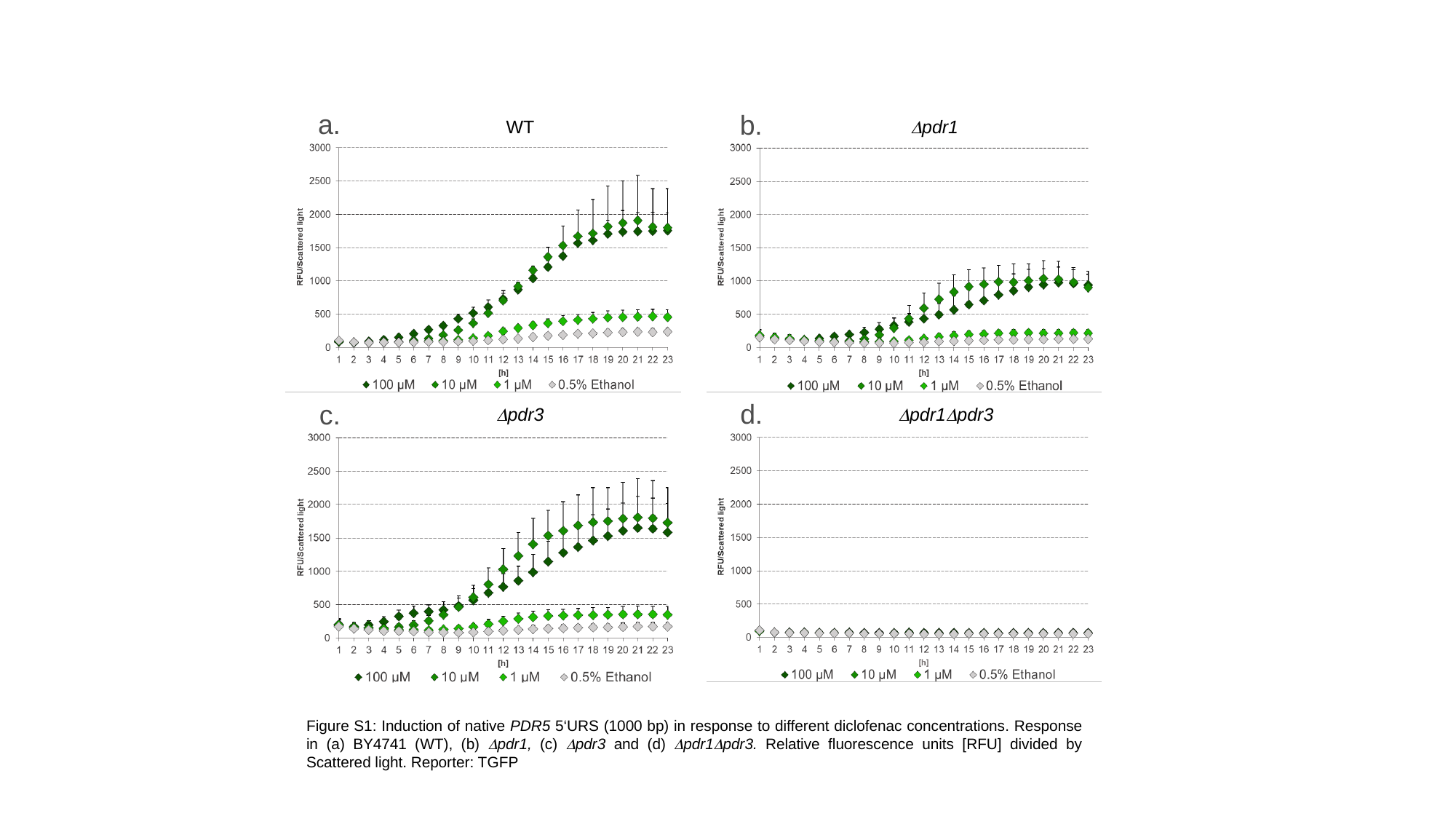

a.
b.
WT
Dpdr1
d.
c.
Dpdr3
Dpdr1Dpdr3
Figure S1: Induction of native PDR5 5‘URS (1000 bp) in response to different diclofenac concentrations. Response in (a) BY4741 (WT), (b) Dpdr1, (c) Dpdr3 and (d) Dpdr1Dpdr3. Relative fluorescence units [RFU] divided by Scattered light. Reporter: TGFP

## Slide 3
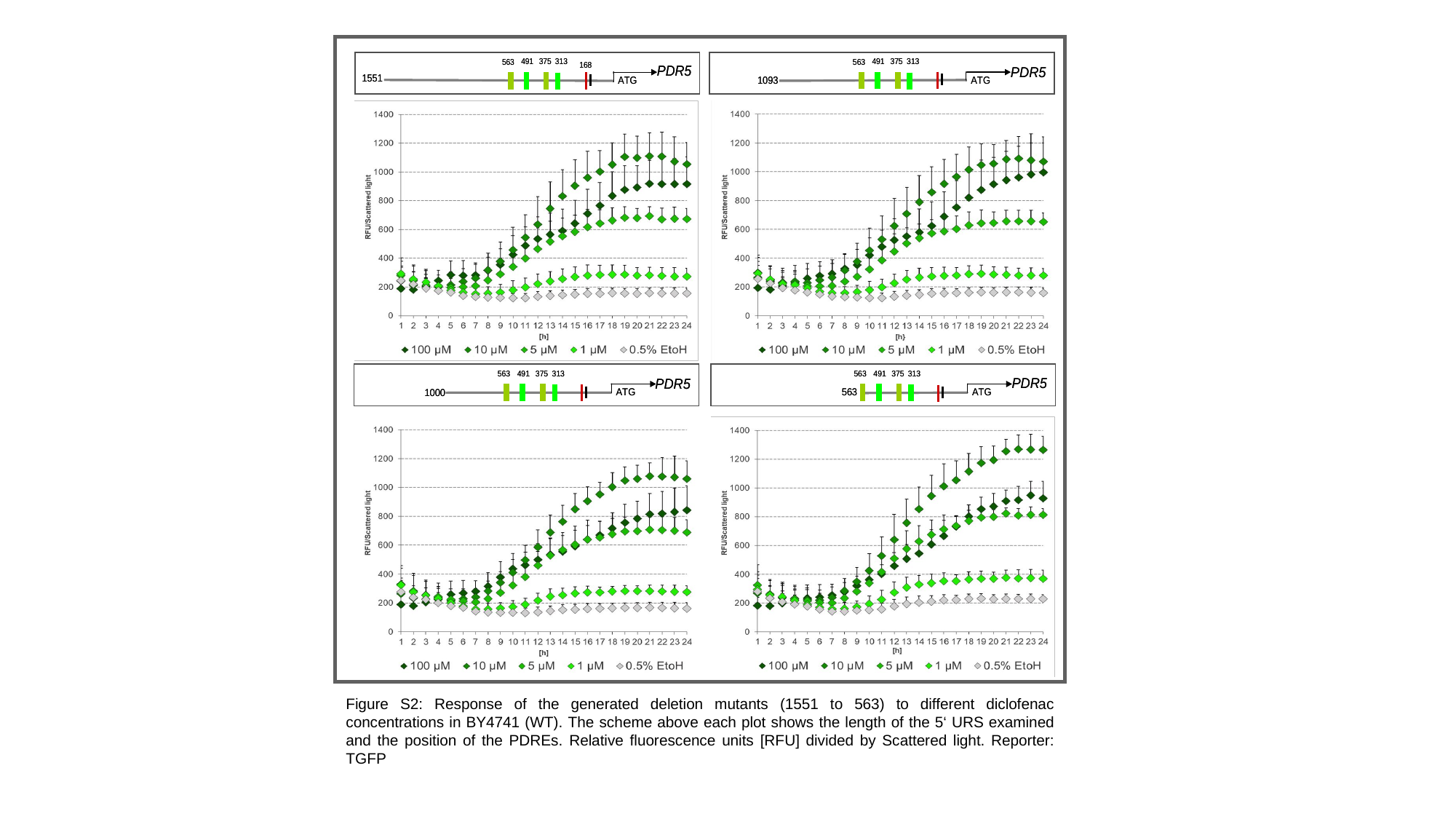

Figure S2: Response of the generated deletion mutants (1551 to 563) to different diclofenac concentrations in BY4741 (WT). The scheme above each plot shows the length of the 5‘ URS examined and the position of the PDREs. Relative fluorescence units [RFU] divided by Scattered light. Reporter: TGFP

## Slide 4
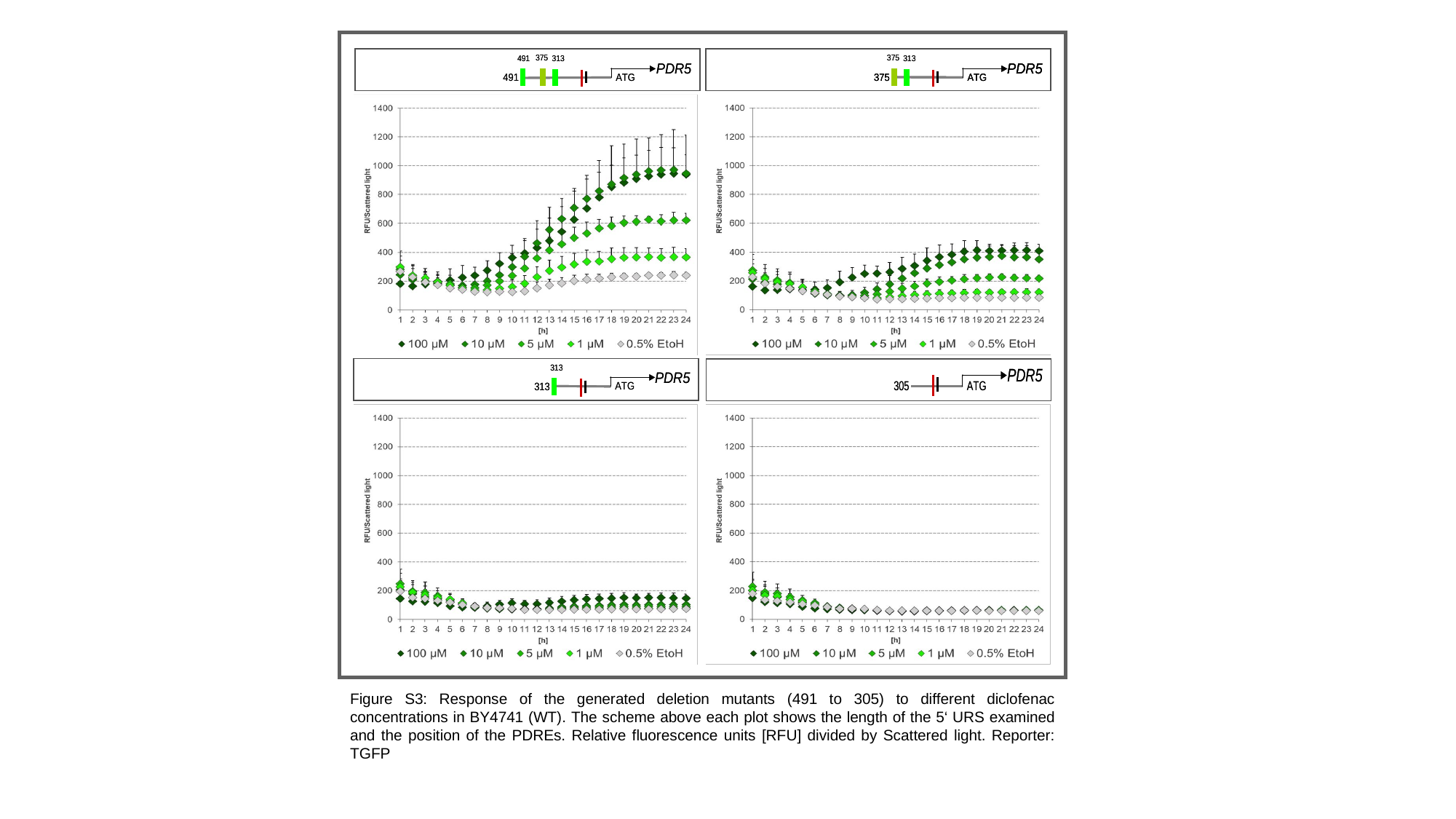

Figure S3: Response of the generated deletion mutants (491 to 305) to different diclofenac concentrations in BY4741 (WT). The scheme above each plot shows the length of the 5‘ URS examined and the position of the PDREs. Relative fluorescence units [RFU] divided by Scattered light. Reporter: TGFP

## Slide 5
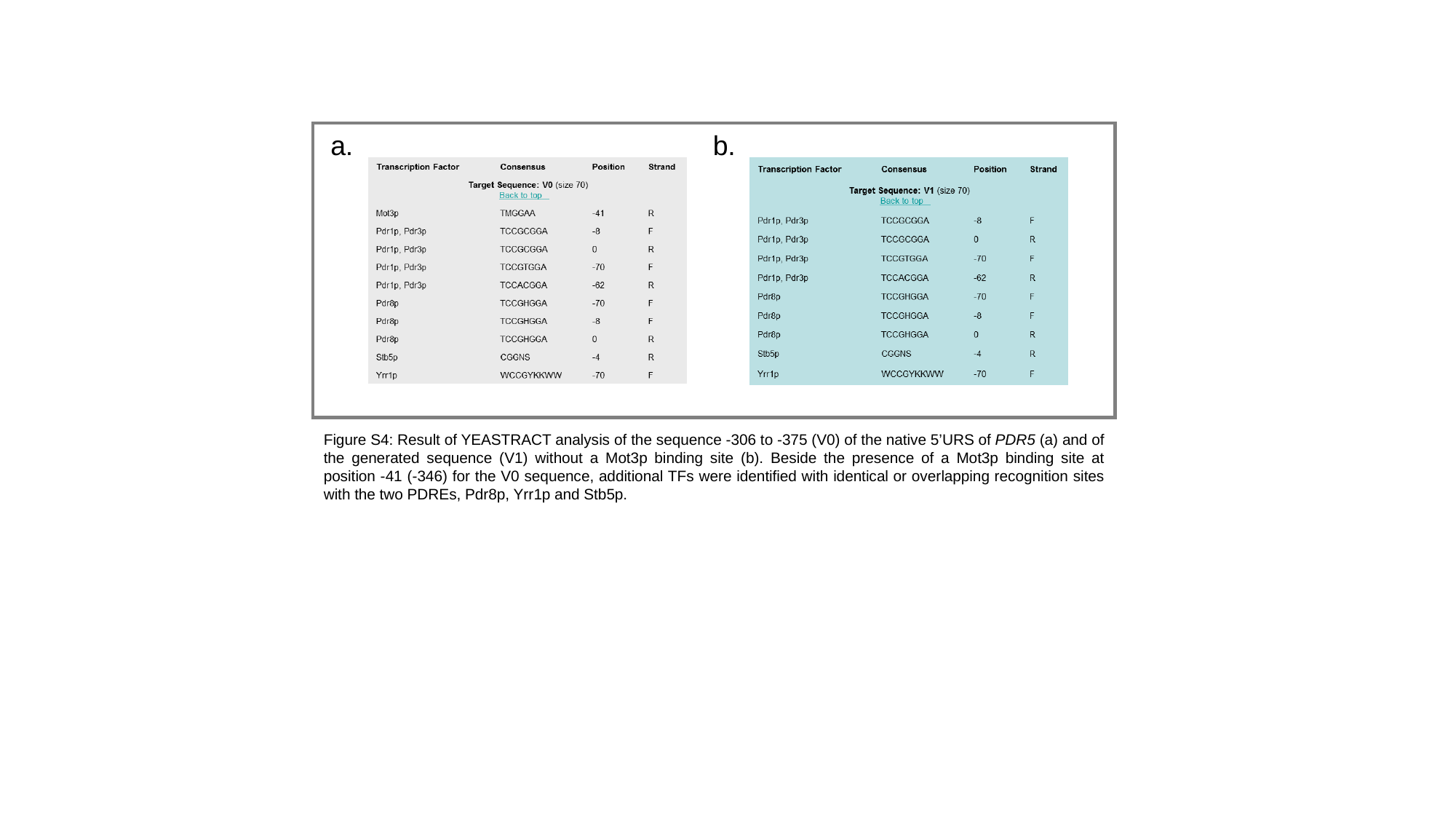

b.
a.
Figure S4: Result of YEASTRACT analysis of the sequence -306 to -375 (V0) of the native 5’URS of PDR5 (a) and of the generated sequence (V1) without a Mot3p binding site (b). Beside the presence of a Mot3p binding site at position -41 (-346) for the V0 sequence, additional TFs were identified with identical or overlapping recognition sites with the two PDREs, Pdr8p, Yrr1p and Stb5p.

## Slide 6
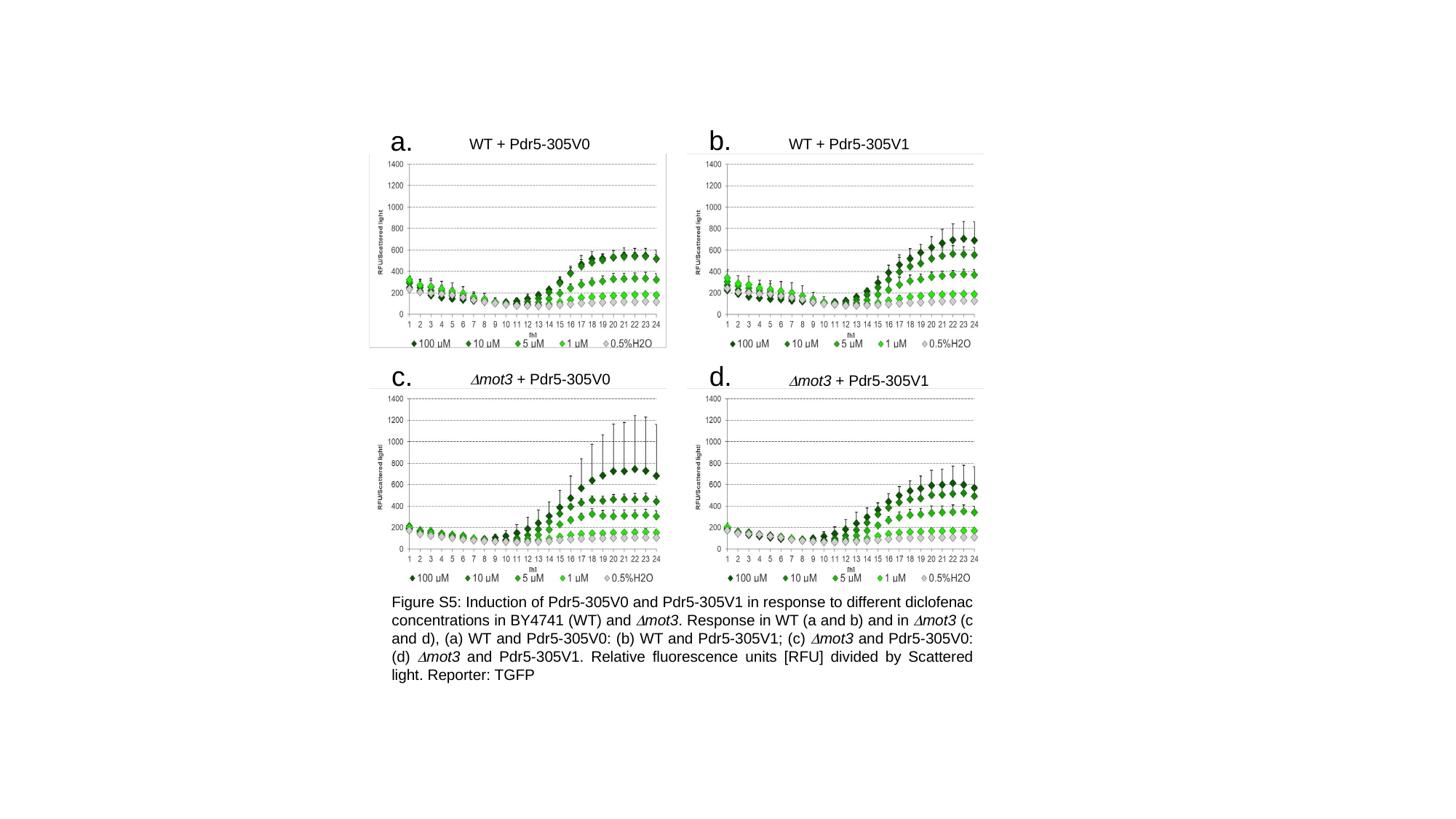

b.
a.
WT + Pdr5-305V0
WT + Pdr5-305V1
c.
d.
Dmot3 + Pdr5-305V0
Dmot3 + Pdr5-305V1
Figure S5: Induction of Pdr5-305V0 and Pdr5-305V1 in response to different diclofenac concentrations in BY4741 (WT) and Dmot3. Response in WT (a and b) and in Dmot3 (c and d), (a) WT and Pdr5-305V0: (b) WT and Pdr5-305V1; (c) Dmot3 and Pdr5-305V0: (d) Dmot3 and Pdr5-305V1. Relative fluorescence units [RFU] divided by Scattered light. Reporter: TGFP

## Slide 7
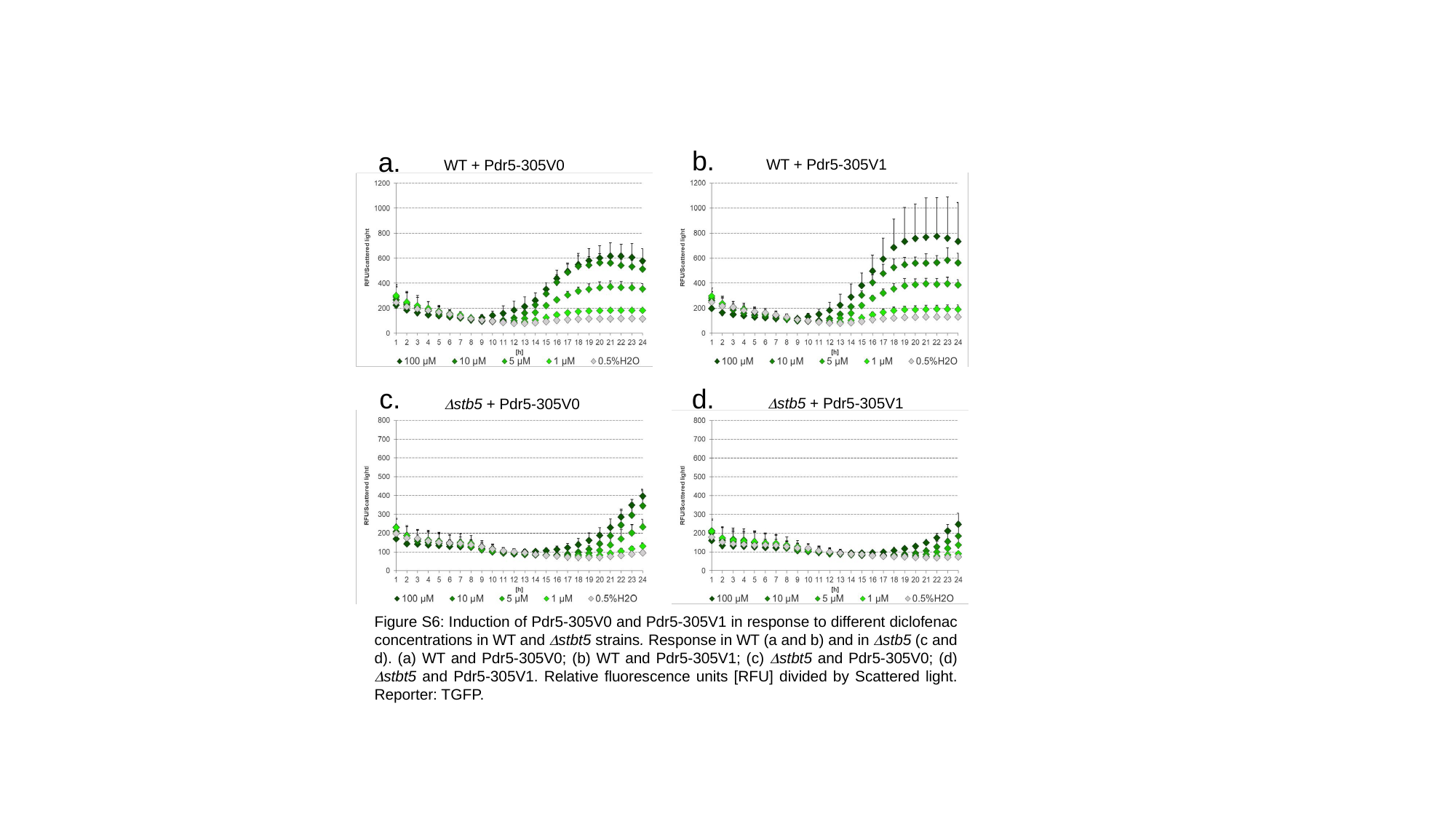

b.
a.
WT + Pdr5-305V1
WT + Pdr5-305V0
c.
d.
Dstb5 + Pdr5-305V1
Dstb5 + Pdr5-305V0
Figure S6: Induction of Pdr5-305V0 and Pdr5-305V1 in response to different diclofenac concentrations in WT and Dstbt5 strains. Response in WT (a and b) and in Dstb5 (c and d). (a) WT and Pdr5-305V0; (b) WT and Pdr5-305V1; (c) Dstbt5 and Pdr5-305V0; (d) Dstbt5 and Pdr5-305V1. Relative fluorescence units [RFU] divided by Scattered light. Reporter: TGFP.

## Slide 8
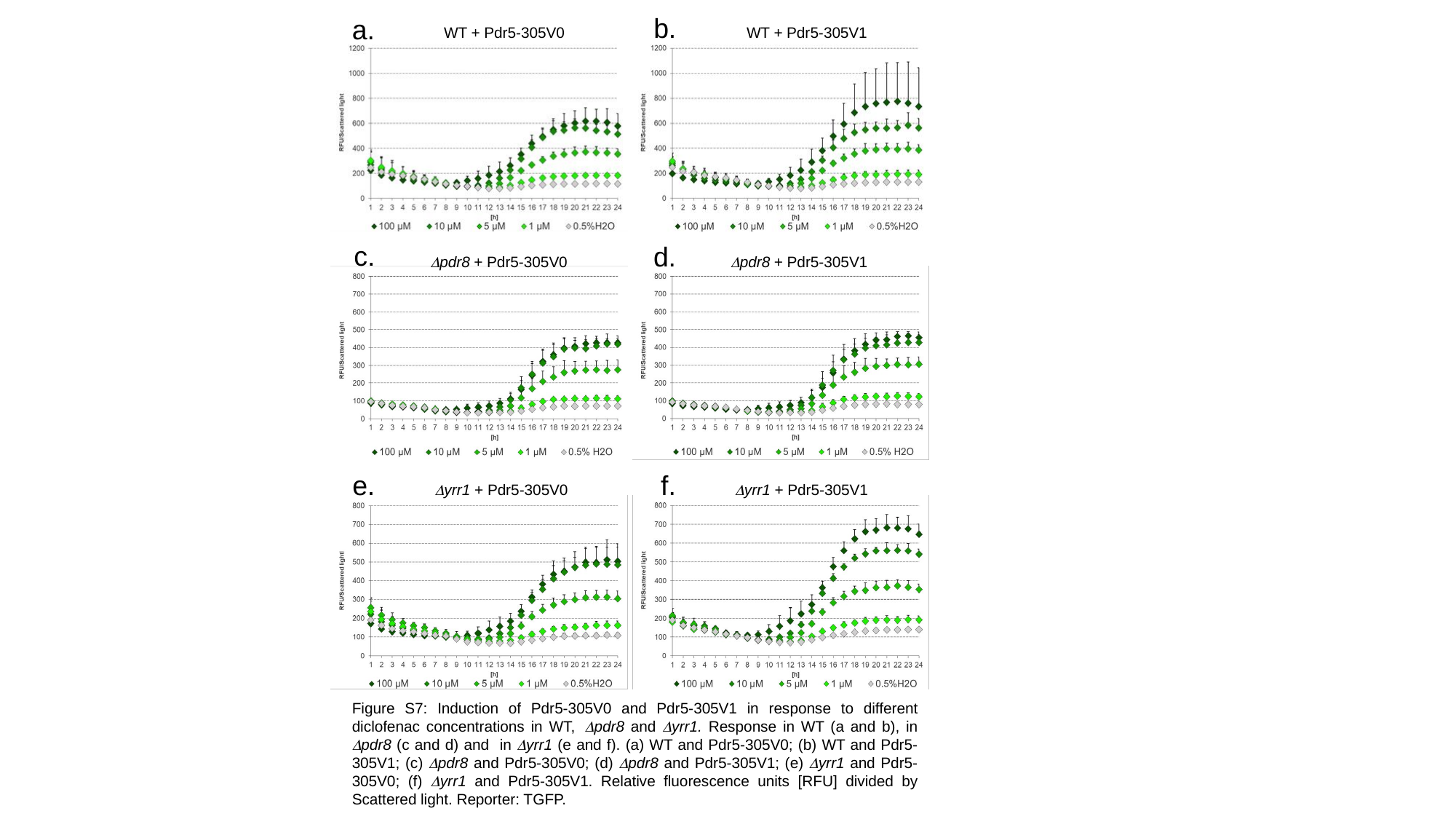

b.
a.
WT + Pdr5-305V0
WT + Pdr5-305V1
c.
d.
Dpdr8 + Pdr5-305V1
Dpdr8 + Pdr5-305V0
f.
e.
Dyrr1 + Pdr5-305V1
Dyrr1 + Pdr5-305V0
Figure S7: Induction of Pdr5-305V0 and Pdr5-305V1 in response to different diclofenac concentrations in WT, Dpdr8 and Dyrr1. Response in WT (a and b), in Dpdr8 (c and d) and in Dyrr1 (e and f). (a) WT and Pdr5-305V0; (b) WT and Pdr5-305V1; (c) Dpdr8 and Pdr5-305V0; (d) Dpdr8 and Pdr5-305V1; (e) Dyrr1 and Pdr5-305V0; (f) Dyrr1 and Pdr5-305V1. Relative fluorescence units [RFU] divided by Scattered light. Reporter: TGFP.

## Slide 9
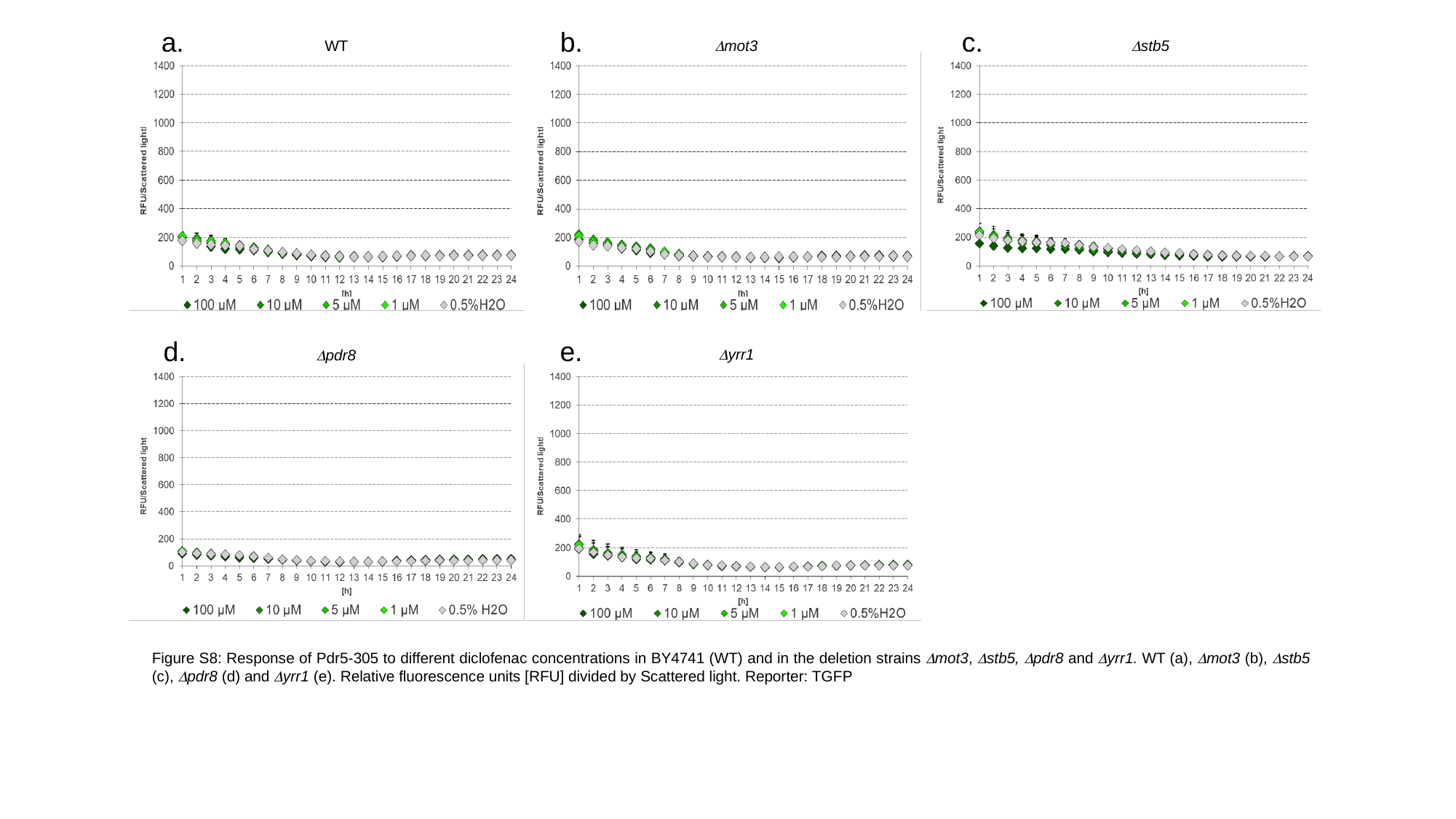

a.
c.
b.
Dstb5
Dmot3
WT
d.
e.
Dyrr1
Dpdr8
Figure S8: Response of Pdr5-305 to different diclofenac concentrations in BY4741 (WT) and in the deletion strains Dmot3, Dstb5, Dpdr8 and Dyrr1. WT (a), Dmot3 (b), Dstb5 (c), Dpdr8 (d) and Dyrr1 (e). Relative fluorescence units [RFU] divided by Scattered light. Reporter: TGFP

## Slide 10
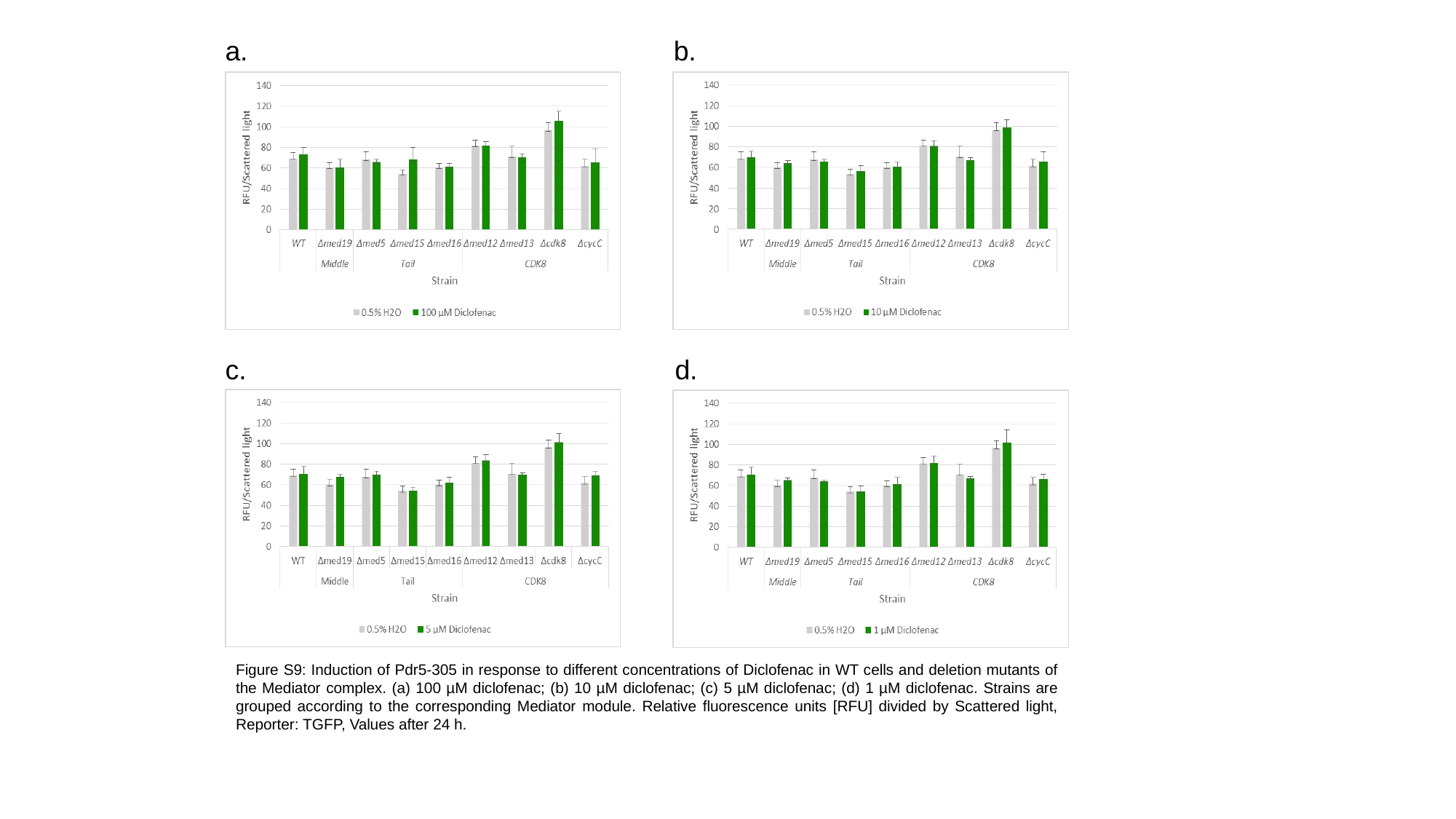

b.
a.
c.
d.
Figure S9: Induction of Pdr5-305 in response to different concentrations of Diclofenac in WT cells and deletion mutants of the Mediator complex. (a) 100 µM diclofenac; (b) 10 µM diclofenac; (c) 5 µM diclofenac; (d) 1 µM diclofenac. Strains are grouped according to the corresponding Mediator module. Relative fluorescence units [RFU] divided by Scattered light, Reporter: TGFP, Values after 24 h.

## Slide 11
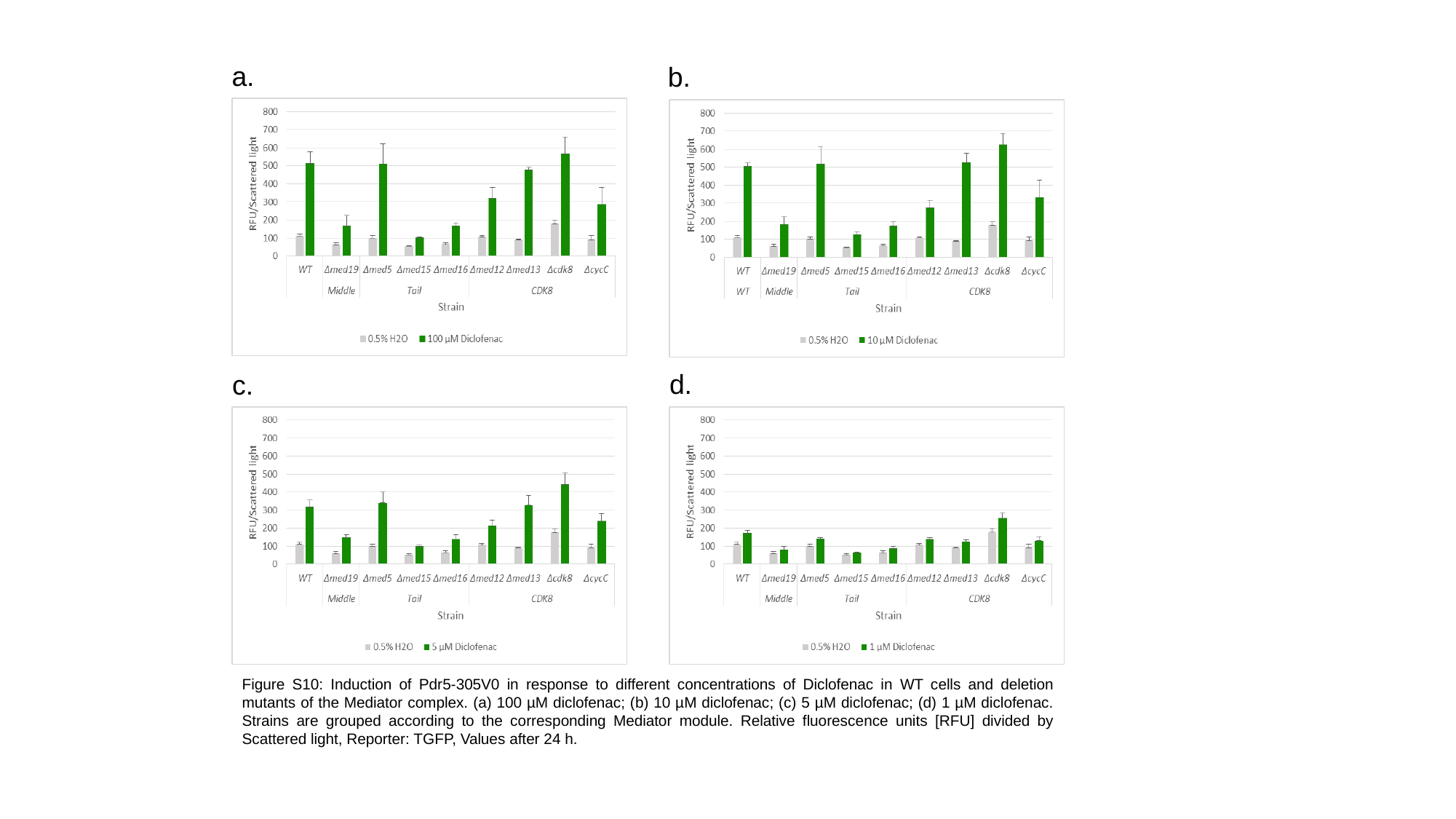

a.
b.
d.
c.
Figure S10: Induction of Pdr5-305V0 in response to different concentrations of Diclofenac in WT cells and deletion mutants of the Mediator complex. (a) 100 µM diclofenac; (b) 10 µM diclofenac; (c) 5 µM diclofenac; (d) 1 µM diclofenac. Strains are grouped according to the corresponding Mediator module. Relative fluorescence units [RFU] divided by Scattered light, Reporter: TGFP, Values after 24 h.

## Slide 12
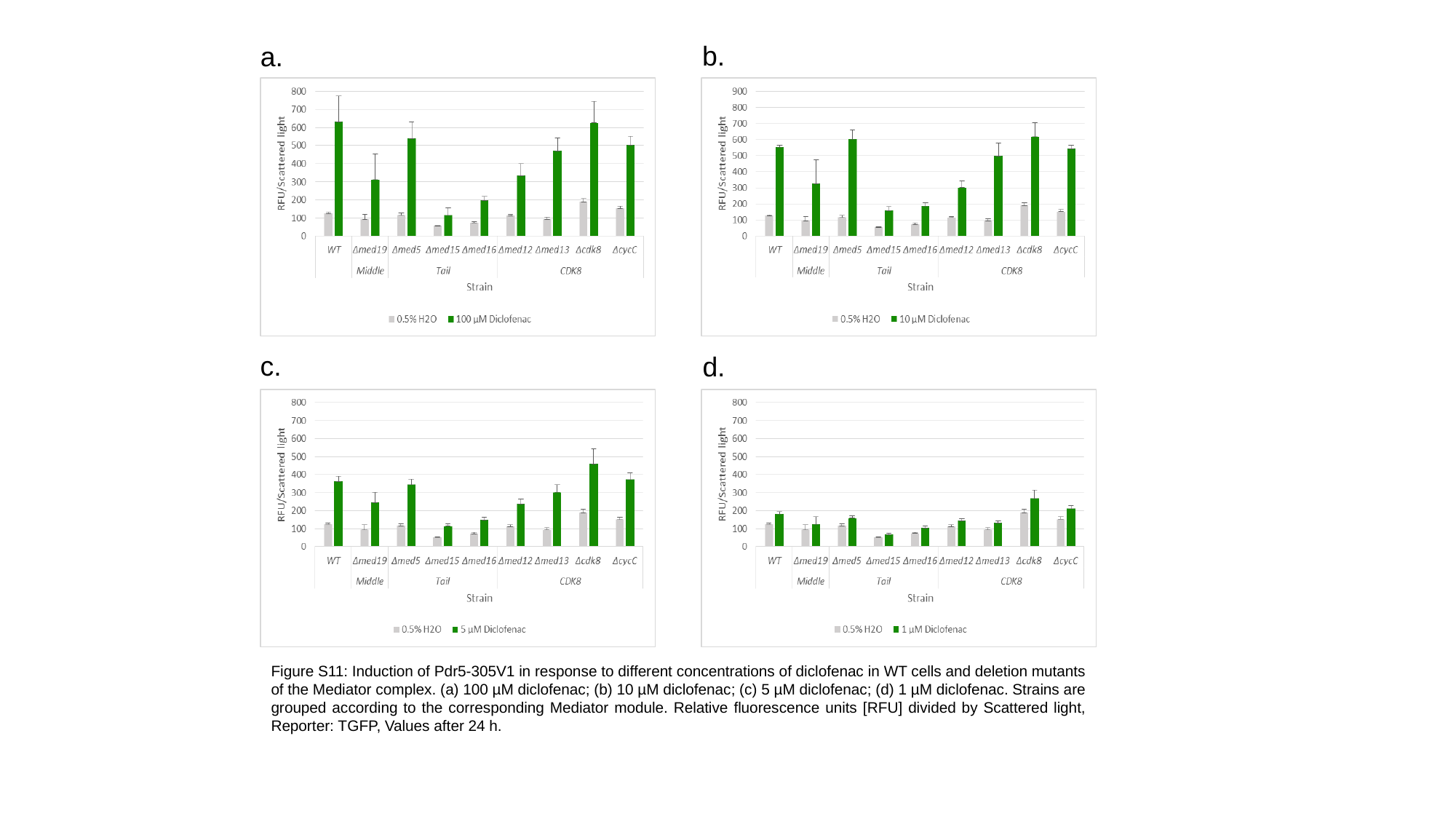

b.
a.
c.
d.
Figure S11: Induction of Pdr5-305V1 in response to different concentrations of diclofenac in WT cells and deletion mutants of the Mediator complex. (a) 100 µM diclofenac; (b) 10 µM diclofenac; (c) 5 µM diclofenac; (d) 1 µM diclofenac. Strains are grouped according to the corresponding Mediator module. Relative fluorescence units [RFU] divided by Scattered light, Reporter: TGFP, Values after 24 h.

## Slide 13
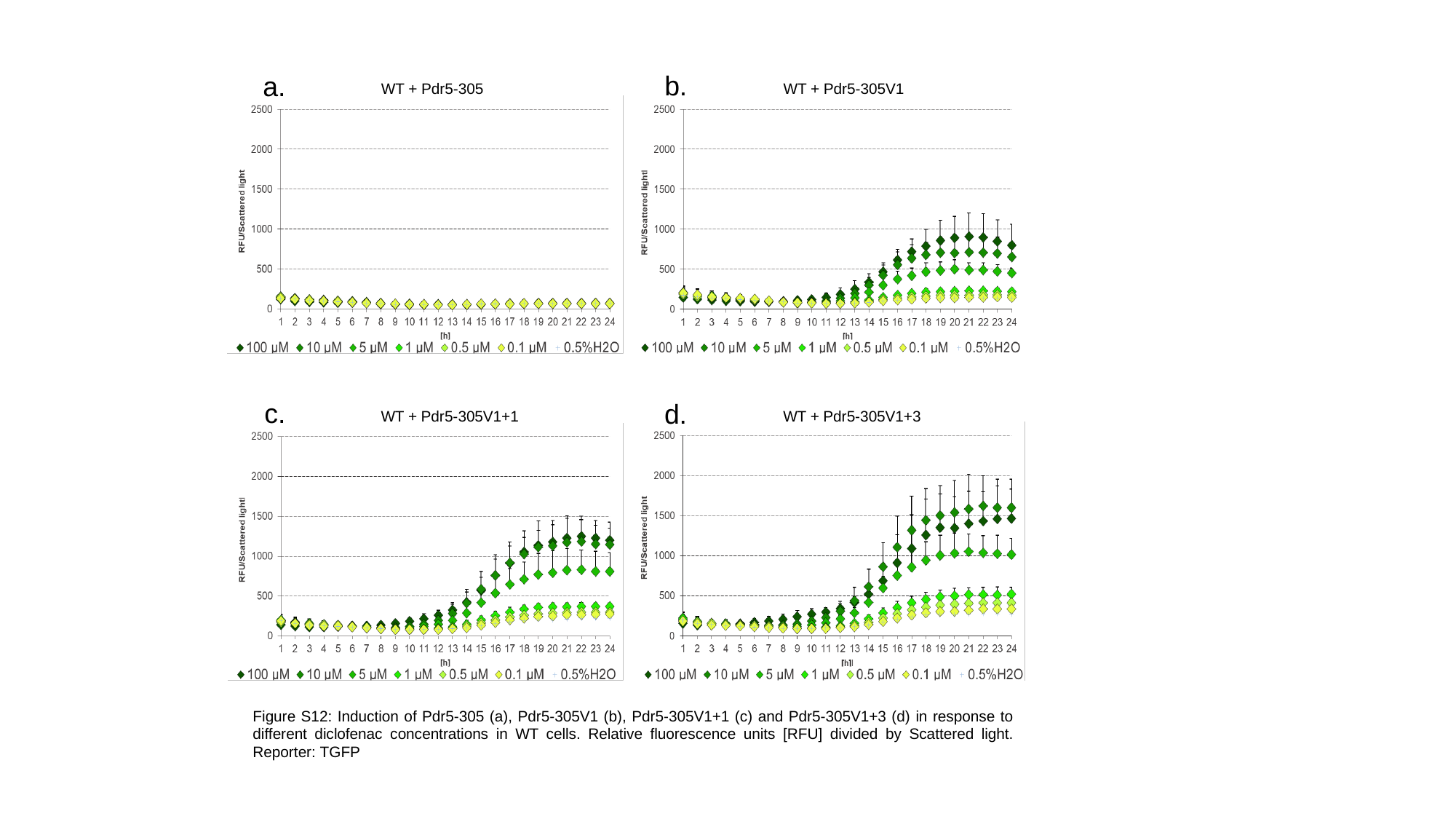

b.
a.
WT + Pdr5-305V1
WT + Pdr5-305
c.
d.
WT + Pdr5-305V1+1
WT + Pdr5-305V1+3
Figure S12: Induction of Pdr5-305 (a), Pdr5-305V1 (b), Pdr5-305V1+1 (c) and Pdr5-305V1+3 (d) in response to different diclofenac concentrations in WT cells. Relative fluorescence units [RFU] divided by Scattered light. Reporter: TGFP
